# Comparative Analysis of Colon Cancer-Derived Fusobacterium nucleatum Subspecies: Inflammation and Colon Tumorigenesis in Murine Models

**DOI:** 10.1128/mbio.02991-21

**Published:** 2022-02-08

**Authors:** Jessica Queen, Jada C. Domingue, James Robert White, Courtney Stevens, Barath Udayasuryan, Tam T. D. Nguyen, Shaoguang Wu, Hua Ding, Hongni Fan, Madison McMann, Alina Corona, Tatianna C. Larman, Scott S. Verbridge, Franck Housseau, Daniel J. Slade, Julia L. Drewes, Cynthia L. Sears

**Affiliations:** a Department of Medicine, Johns Hopkins University grid.471401.7grid.21107.35grid.471401.7grid.21107.35 , Baltimore, Maryland, USA; b Resphera Biosciences, Baltimore, Maryland, USA; c Department of Biomedical Engineering and Mechanics, Virginia Polytechnic Institute, Blacksburg, Virginia, USA; d Department of Biochemistry, Virginia Polytechnic Institute, Blacksburg, Virginia, USA; e Department of Molecular Microbiology and Immunology, Johns Hopkins Bloomberg School of Public Health, Baltimore, Maryland, USA; f Department of Oncology, Johns Hopkins University grid.471401.7grid.21107.35grid.471401.7grid.21107.35 , Baltimore, Maryland, USA; g Division of Gastrointestinal and Liver Pathology, Johns Hopkins University grid.471401.7grid.21107.35grid.471401.7grid.21107.35 , Baltimore, Maryland, USA; h Bloomberg-Kimmel Institute, Johns Hopkins University grid.471401.7grid.21107.35grid.471401.7grid.21107.35 , Baltimore, Maryland, USA; University of Maryland, School of Medicine

**Keywords:** *Fusobacterium* subspecies, colorectal cancer, mouse models, *Fusobacterium* genome sequences, *Fusobacterium* virulence, *Fusobacterium*

## Abstract

Fusobacteria are commonly associated with human colorectal cancer (CRC), but investigations are hampered by the absence of a stably colonized murine model. Further, Fusobacterium nucleatum subspecies isolated from human CRC have not been investigated. While F. nucleatum subspecies are commonly associated with CRC, their ability to induce tumorigenesis and contributions to human CRC pathogenesis are uncertain. We sought to establish a stably colonized murine model and to understand the inflammatory potential and virulence genes of human CRC F. nucleatum, representing the 4 subspecies, *animalis*, *nucleatum*, *polymorphum*, and *vincentii*. Five human CRC-derived and two non-CRC derived F. nucleatum strains were tested for colonization, tumorigenesis, and cytokine induction in specific-pathogen-free (SPF) and/or germfree (GF) wild-type and *Apc^Min/+^* mice, as well as *in vitro* assays and whole-genome sequencing (WGS). SPF wild-type and *Apc^Min/+^* mice did not achieve stable colonization with F. nucleatum, whereas certain subspecies stably colonized some GF mice but without inducing colon tumorigenesis. F. nucleatum subspecies did not form *in vivo* biofilms or associate with the mucosa in mice. *In vivo* inflammation was inconsistent across subspecies, whereas F. nucleatum induced greater cytokine responses in a human colorectal cell line, HCT116. While F. nucleatum subspecies displayed genomic variability, no distinct virulence genes associated with human CRC strains were identified that could reliably distinguish these strains from non-CRC clinical isolates. We hypothesize that the lack of F. nucleatum-induced tumorigenesis in our model reflects differences in human and murine biology and/or a synergistic role for F. nucleatum in concert with other bacteria to promote carcinogenesis.

## INTRODUCTION

Fusobacterium nucleatum is a Gram-negative anaerobe common to the human oral cavity of healthy individuals and those with periodontal disease (1). A heterogenous species consisting of four subspecies, *animalis*, *nucleatum*, *polymorphum*, and *vincentii*, F. nucleatum is the most abundant bacterium in dental plaque biofilms, where it functions as a bridging species to facilitate aggregation and invasion of other bacteria (2–6). F. nucleatum is also the oral organism most commonly associated with nonoropharyngeal diseases, including inflammatory bowel disease, atherosclerosis, organ abscesses, adverse pregnancy outcomes, and, more recently, colorectal cancer (CRC) (1, 7, 8). Clinical studies of North American, European, and Asian cohorts have established that F. nucleatum is enriched in a subset of CRC compared to both paired normal colonic tissues and healthy controls (7–11). What remains unknown is whether F. nucleatum initiates tumor development, promotes tumor progression, or is simply an opportunistic colonizer of the tumor microenvironment.

The association of F. nucleatum with the tumor microenvironment of CRC was first established by several groups using sequencing technologies (7, 8, 12). For instance, Castellarin et al. used RNA sequencing analysis of CRC patients to reveal the overabundance of *Fusobacterium* sequences in tumor tissues compared to matched normal tissues (7). Additionally, two subsequent papers suggested F. nucleatum’s ability to promote and potentiate intestinal tumorigenesis in murine models (13, 14). In one study, daily orogastric inoculation of a Crohn’s disease-derived F. nucleatum isolate (EAVG_002; 7/1) into multiple intestinal neoplasia (*Apc^Min/+^*) mice resulted in modest (median, one colon tumor per *Apc^Min/+^* mouse) induction of colonic tumorigenesis compared to sham or Streptococcus species controls (median, no colon tumors per *Apc^Min/+^* mouse), with tumorigenesis linked to induction of myeloid cell inflammation (13). Further evidence for the role of F. nucleatum in tumorigenesis was demonstrated using an HCT116 cell murine xenograft model (14). Mice injected with purified FadA, one of F. nucleatum’s adhesion factors, showed a 20% increase in xenograft growth compared to mutant FadA protein or bovine serum albumin (BSA) controls, suggesting that FadA contributes to F. nucleatum-associated tumorigenesis.

Additional murine models, as well as *in vitro* investigations with CRC-derived cell lines, have sought to better understand any potential causal role that F. nucleatum plays in CRC (15–21). Studies led by Abed et al. (15, 21) established Fap2 as a lectin, binding to host Gal-GalNAc to mediate F. nucleatum association with colon tumors and CRC cell lines; Fap2 mutants showed reduced binding. Importantly, F. nucleatum localized to tumors via intravenous (i.v.) injection using the CT26 orthotopic mouse model (15). This was supported by a follow-up study directly comparing i.v. injection to oral gavage of F. nucleatum in which intravenously administered F. nucleatum was more successful in tumor colonization (21). Mechanistic insights have implicated Toll-like receptor 4 (TLR4)-β-catenin and TLR4-MYD88 pathways underlying F. nucleatum action in CRC and F. nucleatum induction of microRNAs activating autophagy to promote chemoresistance (16, 19, 20). A few studies support antibacterial therapies for alleviation of F. nucleatum*-*associated CRC. Berberine, an isoquinoline alkaloid used to treat intestinal infections in China, reduced colon tumor numbers in mice inoculated daily with F. nucleatum compared to those with F. nucleatum alone (18). Additionally, metronidazole treatment of a CRC patient-derived xenograft model reduced tumor growth, proliferation, and F. nucleatum tumor load (17).

However, no studies have developed a murine model of stable colonic F. nucleatum colonization, nor have there been comparative analyses of the tumorigenic potential of the F. nucleatum subspecies in existing CRC murine models. To date, which F. nucleatum subspecies may be most relevant to human CRC pathogenesis remains uncertain, although limited data suggest F. nucleatum subspecies *animalis* is more frequently present in CRC tissues (22). Importantly, studies evaluating the effect of long-term daily orogastric gavage with F. nucleatum do not reflect the expected events in human disease in which either passage through the gastrointestinal tract or transient bacteremia facilitates stable colonization of the colon and/or colon tumors. Thus, we tested approaches to develop a murine model of stable intestinal colonization, in contrast to daily oral gavages, while also investigating the inflammatory and tumorigenic potential of several genetically diverse CRC- and non-CRC-derived F. nucleatum subspecies in both mice and the CRC cell line HCT116.

## RESULTS

### CRC-derived F. nucleatum subspecies do not consistently colonize SPF mice.

**FIG 1:**
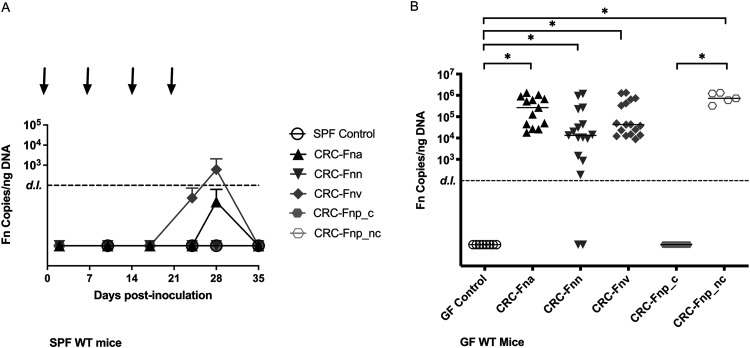
Differential colonization of F. nucleatum subspecies in SPF and GF wild-type (WT) mice. (A) SPF mice were orally gavaged with the designated F. nucleatum subspecies at days 0, 7, 14, and 21 (arrows), and fecal pellets were collected at the indicated time points on the *x* axis. F. nucleatum colonization is plotted as F. nucleatum copies/ng fecal DNA per group over time (mean ± SD), with a detection limit (LOD) of 100 F. nucleatum copies. *n* = 3 control mice and 10 experimental mice per strain. (B) GF WT mice were orally gavaged with F. nucleatum once and then assessed for colonization after 14 days. Each dot indicates the F. nucleatum copies/ng DNA of an individual mouse. Bars indicate the median with interquartile range. (*Fna* denotes F. nucleatum subsp. *animalis*, *Fnn* denotes F. nucleatum subsp. *nucleatum*, *Fnv* denotes F. nucleatum subsp. *vincentii*, *Fnp_c* denotes clumpy F. nucleatum subsp. *polymorphum*, and *Fnp*_*nc* denotes nonclumpy F. nucleatum subsp. *polymorphum*). *n* = 5 to 17 mice/group. One GF control mouse calculated as having 125 F. nucleatum copies/ng DNA was removed from analysis.

While several studies have investigated the role that F. nucleatum plays in gut inflammation and tumorigenesis, those studies failed to report stable colonization of F. nucleatum, and the reported pathophysiological changes were dependent on daily F. nucleatum gavages (13, 16, 18, 23, 24). Additionally, these studies did not investigate CRC-derived F. nucleatum strains. Herein, we used F. nucleatum strains isolated from human CRC tumor biopsy specimens, representing each of the subspecies and from diverse geographic locations (Table 1), and assessed whether CRC-derived F. nucleatum colonized the mouse gut, using antibiotic-treated specific-pathogen-free (SPF) C57BL/6J wild-type (WT) mice inoculated weekly with CRC-derived F. nucleatum strains. Despite repeated orogastric inoculations, all F. nucleatum isolates failed to establish consistent colonic colonization (Fig. 1A). Only mice gavaged with the CRC-derived F. nucleatum subsp. *vincentii* (CRC-F. nucleatum subsp. *vincentii*) reached levels above the limit of detection (LOD), but only following the fourth and final gavage; this signal was quickly lost by 12 days after the final inoculation. Further, mice gavaged with CRC-F. nucleatum subsp. *vincentii* failed to exhibit weight loss or changes in colon length typically seen with inflammation/colitis (25) (data not shown). Because data suggest that host-gene microbe interactions affect pathogenesis (26), we further tested CRC-F. nucleatum subsp. *vincentii* in SPF *Apc^Min/+^* mice. Despite an intensified orogastric gavage protocol (see Materials and Methods), CRC-F. nucleatum subsp. *vincentii* was unable to colonize the mice (Fig. S1 in the supplemental material). Thus, we conclude that CRC-derived F. nucleatum subspecies do not stably colonize the gut of conventionally raised SPF mice, even with repeated inoculations that are typically not necessary with other enteric pathogens.

**TABLE 1:** F. nucleatum strains used in this study

Strain name	Abbreviated designation	Subspecies	Source (reference)
F. nucleatum 173CP^*a*^	CRC-F. nucleatum subsp. *animalis*	*animalis*	CRC patient, Spanish cohort
CTX3 F. nucleatum 10^*a*^	CRC-F. nucleatum subsp. *nucleatum*	*nucleatum*	CRC patient, Spanish cohort
F. nucleatum 146CP^*a*^	CRC-F. nucleatum subsp. *vincentii*	*vincentii*	CRC patient, Spanish cohort
F. nucleatum 3760T	Clumpy CRC-F. nucleatum subsp. *polymorphum*	*polymorphum*	CRC patient, US cohort
F. nucleatum S043-1	Nonclumpy CRC-F. nucleatum subsp. *polymorphum*	*polymorphum*	CRC patient from Malaysian cohort
F. nucleatum EAVG_002^*c*^	Non-CRC-F. nucleatum subsp. *animalis*	*animalis*	IBD^*b*^ patient (63)
F. nucleatum 23726	Non-CRC-F. nucleatum subsp. *nucleatum*	*nucleatum*	Urogenital tract (ATCC)

a Strain provided by S. Bullman, Fred Hutchinson Cancer Research Center, Seattle, WA.

b IBD, irritable bowel disease.

c *F. nucleatum* EAVG_002 is also known as *F. nucleatum* 7/1.

10.1128/mBio.02991-21.4 FIG S1 SPF *Apc^Min/+^* mice were orally gavaged with the F. nucleatum subsp. *vincentii* at days 0, 2, 5, 7, 14, 21, 28, 35, 42, 49, 56, 63, and 70 (arrows), and fecal pellets were collected at the indicated time points on the x axis. F. nucleatum colonization was assessed by quantitative PCR (qPCR) of the F. nucleatum 16S rRNA gene and plotted as the average of F. nucleatum copies/ng fecal DNA (fDNA) per group (±SD). A detection limit (LOD; 100 F. nucleatum copies) was set based on the undetermined *C_T_* value of the F. nucleatum DNA standard. *n* = 2 control mice and 8 F. nucleatum-treated mice. (*Fnv* denotes F. nucleatum subsp. *vincentii*). Download FIG S1, TIF file, 0.3 MB. Copyright © 2022 Queen et al. 2022 Queen et al. https://creativecommons.org/licenses/by/4.0/ This content is distributed under the terms of the Creative Commons Attribution 4.0 International license.

### CRC-derived F. nucleatum subsp. differentially colonize GF mice.

Due to the lack of stable colonization in SPF mice, we hypothesized that, despite the use of microbiome-disrupting antibiotic treatment, F. nucleatum remained unable to overcome competition by the modified microbiota. Thus, we turned to a germfree (GF) murine model. GF wild-type (WT) mice were inoculated once with CRC-derived F. nucleatum isolates, and colonization was assessed after 14 days. We found that F. nucleatum subsp. differed in their ability to colonize GF WT mice (Fig. 1B). In contrast to the SPF model, CRC-derived F. nucleatum subsp. *animalis* (CRC-F. nucleatum subsp. *animalis*), CRC-F. nucleatum subsp. *nucleatum*, and CRC-F. nucleatum subsp. *vincentii* displayed significant colonization levels compared to GF controls (*P* < 0.0001, *P* = 0.001, and *P* < 0.0001, respectively). Notably, a minority of mice (3/17) inoculated with F. nucleatum subsp. *nucleatum* failed to colonize, although those that did were colonized at levels similar to F. nucleatum subsp. *animalis* and F. nucleatum subsp. *vincentii*. Interestingly, the CRC-derived F. nucleatum subsp. *polymorphum* initially tested was unable to colonize the mice. We hypothesized that this may be due to its clumpy, self-aggregative morphology when grown *in vitro* (Fig. S2). We therefore inoculated a group of mice (*n* = 5) with a nonclumpy CRC-derived F. nucleatum subsp. *polymorphum* strain (nonclumpy CRC-F. nucleatum subsp. *polymorphum*), which significantly colonized the mice compared to the clumpy strain (*P* = 0.0005) and GF controls (*P* = 0.0008) and to levels similar to the other isolates; however, it was also unable to colonize SPF WT mice (Fig. 1A).

10.1128/mBio.02991-21.5 FIG S2 *In vitro* growth morphology of each CRC-derived F. nucleatum strain in BHI media. F. nucleatum strains were cultured in BHI medium supplemented with hemin and vitamin K under anaerobic conditions at 37°C for 48 to 72 h in static culture (to an OD_600_ of ∼1.5). (*Fna* denotes F. nucleatum subsp. *animalis*, *Fnn* denotes F. nucleatum subsp. *nucleatum*, *Fnv* denotes F. nucleatum subsp. *vincentii*, *Fnp_c* denotes clumpy F. nucleatum subsp. *polymorphum*, and *Fnp*_*nc* denotes nonclumpy F. nucleatum subsp. *polymorphum*). Download FIG S2, TIF file, 2.6 MB. Copyright © 2022 Queen et al. 2022 Queen et al. https://creativecommons.org/licenses/by/4.0/ This content is distributed under the terms of the Creative Commons Attribution 4.0 International license.

As F. nucleatum contributes to biofilm development of oral dental plaques and is also present in a majority of CRC-associated colonic biofilms (27), we also assessed distal colons for the presence of F. nucleatum biofilms by fluorescent *in situ* hybridization (FISH). Despite stable colonization of most F. nucleatum subsp. in GF WT mice, mucus-invasive biofilms were not found. Only 3/21 mice (14%) evaluated displayed mucosal staining with the all-bacterial or *Fusobacterium*-specific probe (Fig. S3). Thus, while prior data support that F. nucleatum stably colonizes the gut of monognotobiotic mice (28), our colonization data suggest variable colonization potential of F. nucleatum isolates with very limited mucosal association in gnotobiotic mice.

10.1128/mBio.02991-21.6 FIG S3 Fluorescence *in situ* hybridization of GF WT colons at 2 weeks post*-*F. nucleatum inoculation at ×40 magnification. DAPI (4′,6-diamidino-2-phenylindole; blue) and universal bacterial probe (yellow) with F. nucleatum visible in some sections (red arrow). (A) GF control; (B) CRC-F. nucleatum subsp. *animalis*; (C) CRC-F. nucleatum subsp. *nucleatum*; (D) CRC-F. nucleatum subsp. *vincentii*; (E) clumpy CRC-F. nucleatum subsp. *polymorphum*; (F) nonclumpy F. nucleatum subsp. *polymorphum*. Download FIG S3, TIF file, 2.8 MB. Copyright © 2022 Queen et al. 2022 Queen et al. https://creativecommons.org/licenses/by/4.0/ This content is distributed under the terms of the Creative Commons Attribution 4.0 International license.

### CRC-derived F. nucleatum subspecies do not differ in induction of proinflammatory gene expression in GF WT mice.

**FIG 2:**
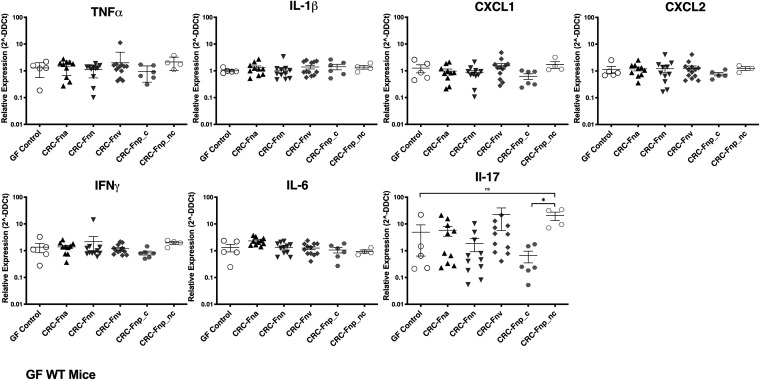
Changes in distal colon inflammatory gene expression. GF WT mice were inoculated with the indicated F. nucleatum strains then harvested after 14 days. RNA was extracted from distal colon tissue, and relative quantification of gene expression was performed with qRT-PCR using TaqMan gene expression Assays for each target gene, normalized to murine GAPDH. Data are plotted as the relative expression (threshold cycle [2^−ΔΔ^*^CT^*]) per mouse. Bars indicate the median with interquartile range. *n* = 5 to 17 mice/group. (*Fna* denotes F. nucleatum subsp. *animalis*, *Fnn* denotes F. nucleatum subsp. *nucleatum*, *Fnv* denotes F. nucleatum subsp. *vincentii*, *Fnp_c* denotes clumpy F. nucleatum subsp. *polymorphum*, and *Fnp*_*nc* denotes nonclumpy F. nucleatum subsp. *polymorphum*).

Having demonstrated that GF mice allow for significant colonization of several CRC-derived F. nucleatum strains, we next investigated whether F. nucleatum colonization impacts inflammatory gene expression in the distal colon. Despite robust colonization by F. nucleatum subsp. *animalis*, F. nucleatum subsp. *vincentii*, F. nucleatum subsp. *nucleatum*, and nonclumpy F. nucleatum subsp. *polymorphum* (Fig. 1B), we found no significant changes in expression of several cytokine and chemokine genes in the distal colon compared to GF control mice (Fig. 2). While the majority of genes had very similar expression across groups compared to the GF controls, expression of interleukin 17a (IL-17a) was highly variable within and across groups. Notably, the nonclumpy F. nucleatum subsp. *polymorphum* strain significantly upregulated IL-17a expression in comparison to the clumpy F. nucleatum subsp. *polymorphum* isolate (*P* = 0.0095); however, this still did not differ from the GF controls (*P* = 0.0635). Further, IL-17 levels did not correlate with F. nucleatum copy number (linear regression; *P* = 0.11, *R*^2^ = 0.06). Similar to SPF mice, there were no changes in body weight or colon length in GF WT mice (data not shown).

### F. nucleatum subsp. differ in colonization and induction of proinflammatory gene expression but do not promote tumorigenesis in GF *Apc^Min/+^* mice.

**FIG 3:**
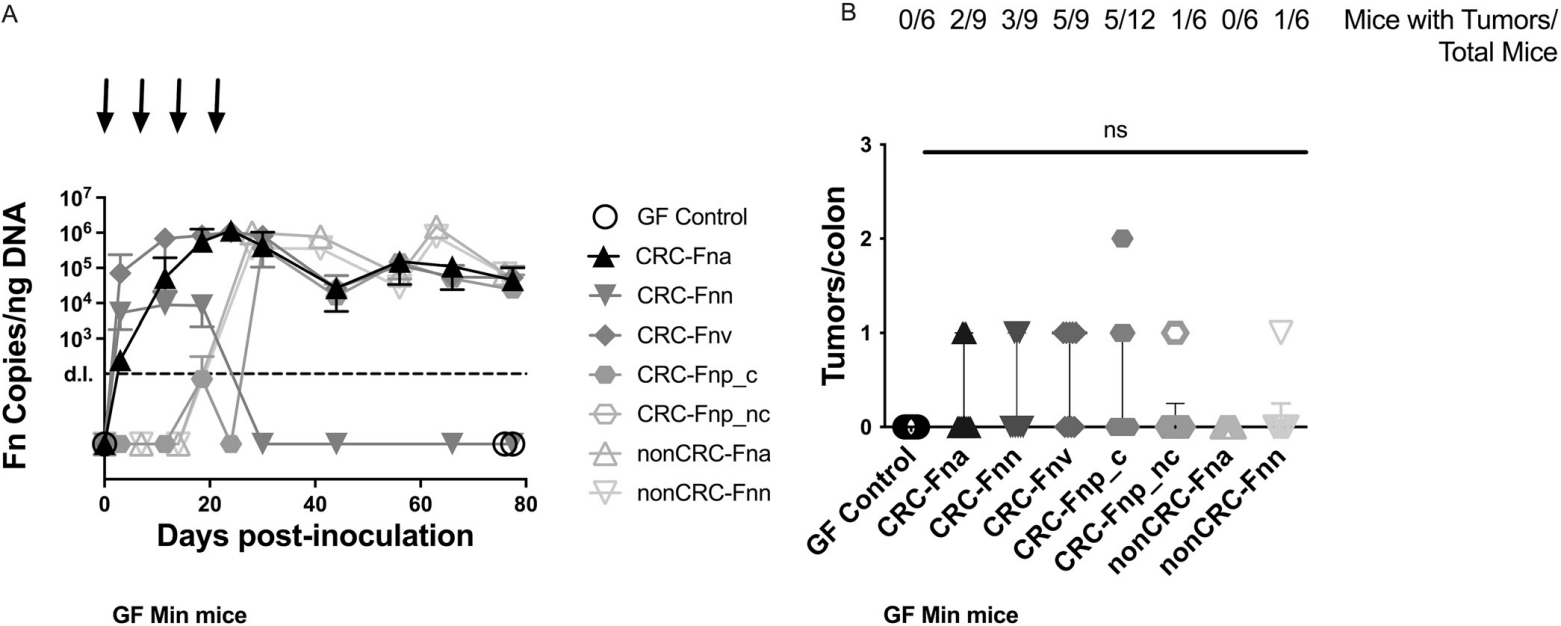
Colonization and tumorigenesis in F. nucleatum-treated GF *Apc^Min/+^* mice. GF *Apc^Min/+^* mice were orally gavaged once per week for 4 weeks (arrows), and fecal pellets were collected at the indicated time points on the *x* axis. (A) Colonization is plotted as F. nucleatum copies/ng fecal DNA per group over time (mean ± SD) with a detection limit (LOD) of 100 F. nucleatum copies. (B) Data are displayed as number of tumors/colon per mouse. Bars indicate the median with interquartile range. The number of mice with tumors out of total mice inoculated with each strain are displayed above the graph. *n* = 6 to 12 mice/group. (*Fna* denotes F. nucleatum subsp. *animalis*, *Fnn* denotes F. nucleatum subsp. *nucleatum*, *Fnv* denotes F. nucleatum subsp. *vincentii*, *Fnp_c* denotes clumpy F. nucleatum subsp. *polymorphum*, and *Fnp*_*nc* denotes nonclumpy F. nucleatum subsp. *polymorphum*).

Since previous studies in murine models showed a strong link between inflammation and colon tumorigenesis (29), we expected that, over time, F. nucleatum would alter the immune microenvironment of mice susceptible to intestinal tumorigenesis and promote colon tumors. For these experiments, GF *Apc^Min/+^* mice were gavaged weekly for 4 weeks. Similar to our findings in GF WT mice (Fig. 1B), we observed differences in colonization of GF *Apc^Min/+^* mice (Fig. 3A), with delayed uptake of the clumpy F. nucleatum subsp. *polymorphum* strain and lack of persistent colonization in mice inoculated with the CRC-F. nucleatum subsp. *nucleatum* strain. Numerous mice gavaged with each subspecies were able to clear F. nucleatum after weekly inoculations ceased; only 47% (24/51 mice) remained colonized at the end of the 11-week experiments. In contrast to previous studies with daily inoculation of F. nucleatum in SPF *Apc^MinΔ850/+^* mice (13), but similar to studies in gnotobiotic *Apc^MinΔ850/+^* mice inoculated weekly with F. nucleatum (28), we found that stable colonization of GF *Apc^Min/+^* mice with CRC-derived F. nucleatum strains was not associated with an increase in colon tumors (Fig. 3B). Overall, 1 tumor was detected in 17 of 57 (30%) of GF *Apc^Min/+^* mice gavaged with F. nucleatum subsp., and only 1 mouse displayed 2 tumors. Further, when we tested two non-CRC-derived F. nucleatum subsp. *animalis* and F. nucleatum subsp. *nucleatum* strains previously shown to have proinflammatory and protumorigenic effects (Table 1) (13, 15, 30), they were also unable to significantly induce colon tumorigenesis (Fig. 3B). Surprisingly, mice persistently colonized with F. nucleatum were less likely to have a colon tumor (5/30 mice; 17%) than mice that cleared F. nucleatum (12/27 mice; 44%) (*P* = 0.04) (Fig. S4). Parallel to our findings in WT mice, there were no changes in body weight or colon length in F. nucleatum-colonized GF *Apc^Min/+^* mice (Fig. S5).

10.1128/mBio.02991-21.7 FIG S4 GF *Apc^Min/+^* mice were orally gavaged once per week for 4 weeks with the indicated strains, and distal colons were harvested at 11 weeks. Tumors formation was compared for mice that were persistently colonized with F. nucleatum from all groups versus mice that had cleared F. nucleatum from all groups. *n* = 57 total mice. Download FIG S4, TIF file, 0.1 MB. Copyright © 2022 Queen et al. 2022 Queen et al. https://creativecommons.org/licenses/by/4.0/ This content is distributed under the terms of the Creative Commons Attribution 4.0 International license.

10.1128/mBio.02991-21.8 FIG S5 GF *Apc^Min/+^* mice were orally gavaged once per week for 4 weeks with the indicated strains, and distal colons were harvested at 11 weeks. (A and B) Body weight in grams (A) and colon length in centimeters (B) depicted for each mouse with errors bars depicting medians with interquartile range. (C) Representative hematoxylin and eosin (H&E)-stained sections of distal colon, ×20. *n* = 6 to 12 mice/group. Mice were only included in the histopathologic analysis if they remained stably colonized for the duration of the experiment (Therefore, strain CRC-F. nucleatum subsp. *nucleatum* is excluded from this panel). (*Fna* denotes F. nucleatum subsp. *animalis*, *Fnn* denotes F. nucleatum subsp. *nucleatum*, *Fnv* denotes F. nucleatum subsp. *vincentii*, *Fnp_c* denotes clumpy F. nucleatum subsp. *polymorphum*, and *Fnp*_*nc* denotes nonclumpy F. nucleatum subsp. *polymorphum*). Download FIG S5, TIF file, 0.4 MB. Copyright © 2022 Queen et al. 2022 Queen et al. https://creativecommons.org/licenses/by/4.0/ This content is distributed under the terms of the Creative Commons Attribution 4.0 International license.

**FIG 4:**
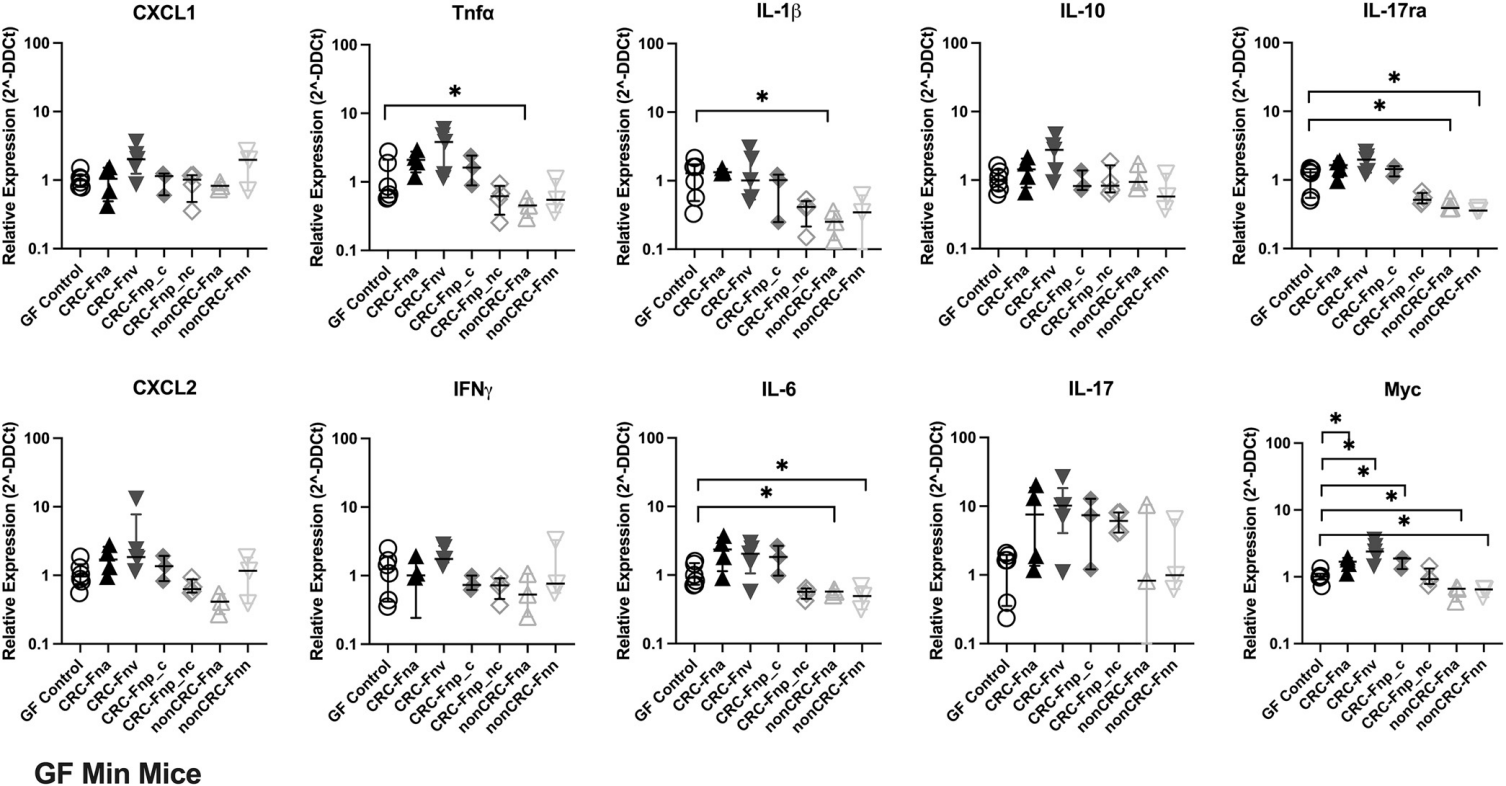
Changes in distal colon inflammatory gene expression in GF *Apc^Min/+^* mice. GF *Apc^Min/+^* mice were orally gavaged once per week for 4 weeks with the indicated strains, and distal colons were harvested at 11 weeks. Mice were only included in the analysis if they remained stably colonized for the duration of the experiment. (Therefore, strain CRC-F. nucleatum subsp. *nucleatum* is excluded from this figure.) Data are plotted as the relative expression (2^−ΔΔ^*^CT^*) per mouse. Bars indicate the median with interquartile range. All *P* values of <0.05 were considered significant. *n* = 3 to 6 mice/group. (*Fna* denotes F. nucleatum subsp. *animalis*, *Fnn* denotes F. nucleatum subsp. *nucleatum*, *Fnv* denotes F. nucleatum subsp. *vincentii*, *Fnp_c* denotes clumpy F. nucleatum subsp. *polymorphum*, and *Fnp*_*nc* denotes nonclumpy F. nucleatum subsp. *polymorphum*).

Evaluation of distal colon gene expression in GF *Apc^Min/+^* mice stably colonized with F. nucleatum by TaqMan Array, covering 45 different genes, revealed that, with the exception of nonclumpy *CRC-*F. nucleatum subsp. *polymorphum*, the CRC-derived F. nucleatum strains modestly but significantly upregulated expression of a number of proinflammatory cytokine and chemokine genes compared to GF control mice (Fig. 4; Table S2). There was little consistency in the genes upregulated by different CRC-derived F. nucleatum strains, with the exception of *Myc* (myelocytomatosis oncogene), which was upregulated by CRC-F. nucleatum subsp. *animalis*, CRC-F. nucleatum subsp. *vincentii*, and CRC-F. nucleatum subsp. *polymorphum*, and conversely downregulated by both non-CRC-derived F. nucleatum strains. Interestingly, the non-CRC strains also downregulated IL-6, IL-1β, and IL-17ra, suggesting a potential impact on type 3 immune cell function. Furthermore, histopathological analysis of GF *Apc^Min/+^* mice revealed no significant mucosal injury or inflammation; a subset of both F. nucleatum-colonized and control mice displayed mild reactive changes and mild crypt hyperplasia (<2×, 9/23 mice) (Fig. S5).

10.1128/mBio.02991-21.3 TABLE S2 Description and results of TaqMan gene assays performed on GF *Apc^Min/+^* mouse distal colons 11 weeks after gavage with F. nucleatum (key genes depicted in Fig. 4). Columns include TaqMan gene assay, gene symbol, gene name, median relative gene expression per group (2^−ΔΔ^*^CT^*), and *P* values calculated by Mann-Whitney U test in comparison to GF control mice. Significant *P* values (<0.05) are bold. Upregulated genes are depicted in red, and downregulated genes are depicted in blue. (*Fna* denotes F. nucleatum subsp. *animalis*, *Fnn* denotes F. nucleatum subsp. *nucleatum*, *Fnv* denotes F. nucleatum subsp. *vincentii*, *Fnp_c* denotes clumpy F. nucleatum subsp. *polymorphum*, and *Fnp*_*nc* denotes nonclumpy F. nucleatum subsp. *polymorphum*). Download Table S2, PDF file, 0.1 MB. Copyright © 2022 Queen et al. 2022 Queen et al. https://creativecommons.org/licenses/by/4.0/ This content is distributed under the terms of the Creative Commons Attribution 4.0 International license.

### CRC-derived F. nucleatum subsp. differentially induce inflammation in a human CRC cell line *in vitro*.

**FIG 5:**
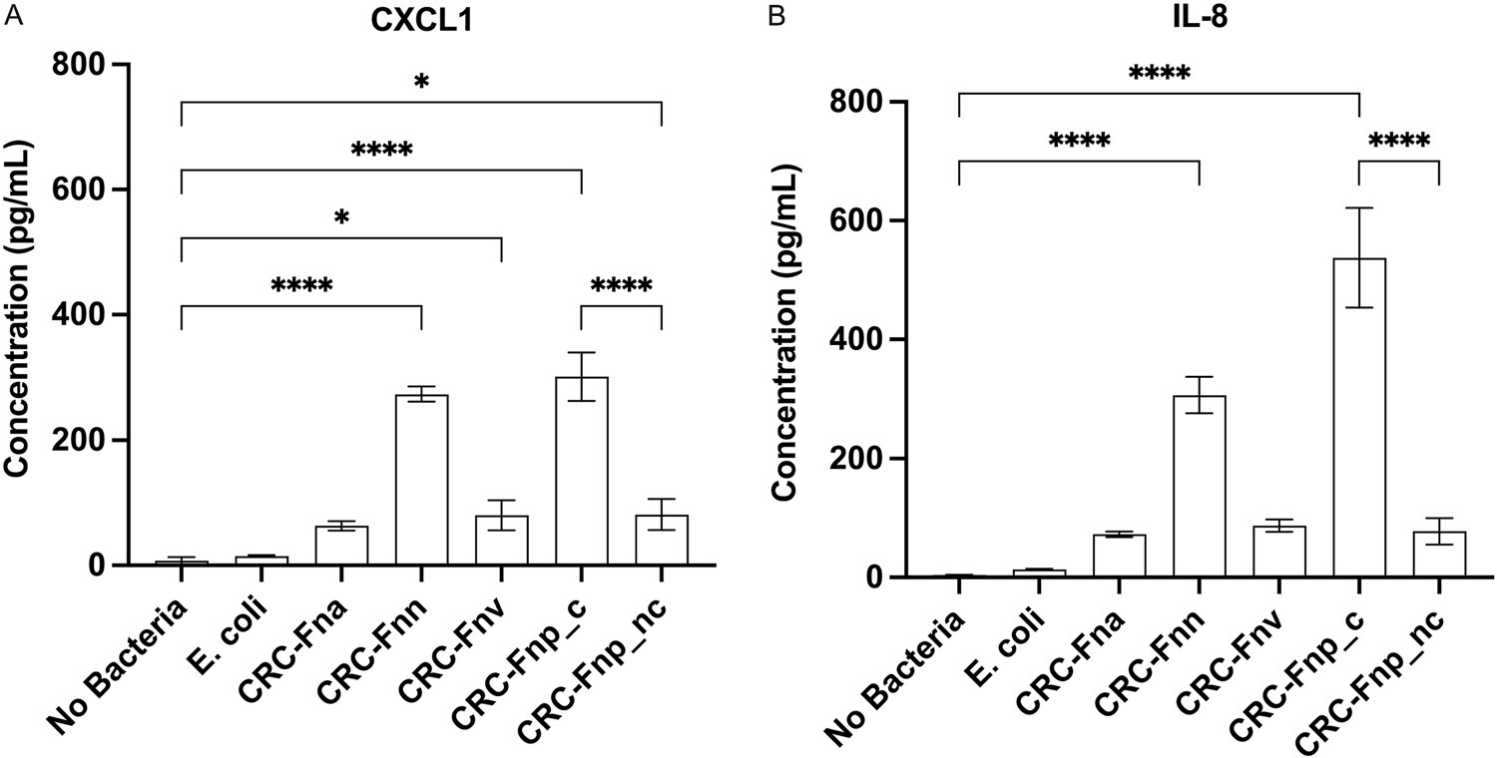
Secretion of CXCL1 (A) and IL-8 (B) from F. nucleatum-treated human HCT116 cells. HCT116 cells were incubated with F. nucleatum strains at an MOI of 50:1 for 4 h, and supernatants were analyzed by ELISA, performed in triplicate. Data are presented as mean ± SD. All strains depicted herein are CRC-derived isolates. For non-CRC isolates, see Fig. S6 in the supplemental material. (*Fna* denotes F. nucleatum subsp. *animalis*, *Fnn* denotes F. nucleatum subsp. *nucleatum*, *Fnv* denotes F. nucleatum subsp. *vincentii*, *Fnp_c* denotes clumpy F. nucleatum subsp. *polymorphum*, and *Fnp*_*nc* denotes nonclumpy F. nucleatum subsp. *polymorphum*).

Because F. nucleatum has been shown to colonize existing human colon tumors and is hypothesized to promote CRC progression by modifying the tumor microenvironment (14), we tested the proinflammatory capacity of our CRC-derived F. nucleatum strains *in vitro* in the human CRC HCT116 cell line. Similar to previous findings with non-CRC-F. nucleatum isolates (30), our CRC- (Fig. 5) and non-CRC-derived (Fig. S6) F. nucleatum strains induced secretion of CXCL1 and IL-8 from HCT116 cells, although the magnitude of chemokine induction differed between strains, even within a given subspecies. While both CRC- and non-CRC-derived F. nucleatum subsp. *nucleatum* strains induced similar levels of chemokine secretion (CXCL1 *P* = 0.9998, IL-8 *P* = 0.9957), the CRC- and non-CRC-derived F. nucleatum subsp. *animalis* strains significantly differed for both chemokines (*P* < 0.0001), with the CRC strains paradoxically inducing less chemokine expression (Fig. S6). Interestingly, the clumpy CRC-derived F. nucleatum subsp. *polymorphum* strain induced significantly more chemokine secretion *in vitro* than the CRC-derived nonclumpy F. nucleatum subsp. *polymorphum* strain (*P* < 0.0001). Notably, the CRC-derived F. nucleatum isolates that most potently induced secretion of CXCL1 and IL-8 from HCT116 cells were those which were least adept at colonizing our GF *Apc^Min/+^* murine model (Fig. 3), highlighting the potential importance of specific host (human versus murine)-microbe interactions.

10.1128/mBio.02991-21.9 FIG S6 CRC and non-CRC-derived F. nucleatum strains differ in their ability to induce secretion of CXCL1 (A) and IL-8 (B) from human HCT116 cells. HCT116 cells were incubated with F. nucleatum strains at an MOI of 50:1 for 4 h, and supernatants were analyzed by ELISA, performed in triplicate. Data are presented as mean ± SD. All *P* values of <0.05 were considered significant (denoted by asterisks). (*Fna* denotes F. nucleatum subsp. *animalis*. *Fnn* denotes F. nucleatum subsp. *nucleatum*. Download FIG S6, TIF file, 0.1 MB. Copyright © 2022 Queen et al. 2022 Queen et al. https://creativecommons.org/licenses/by/4.0/ This content is distributed under the terms of the Creative Commons Attribution 4.0 International license.

### F. nucleatum subsp. differ in genome sequences and copy number of key virulence factors.

**FIG 6:**
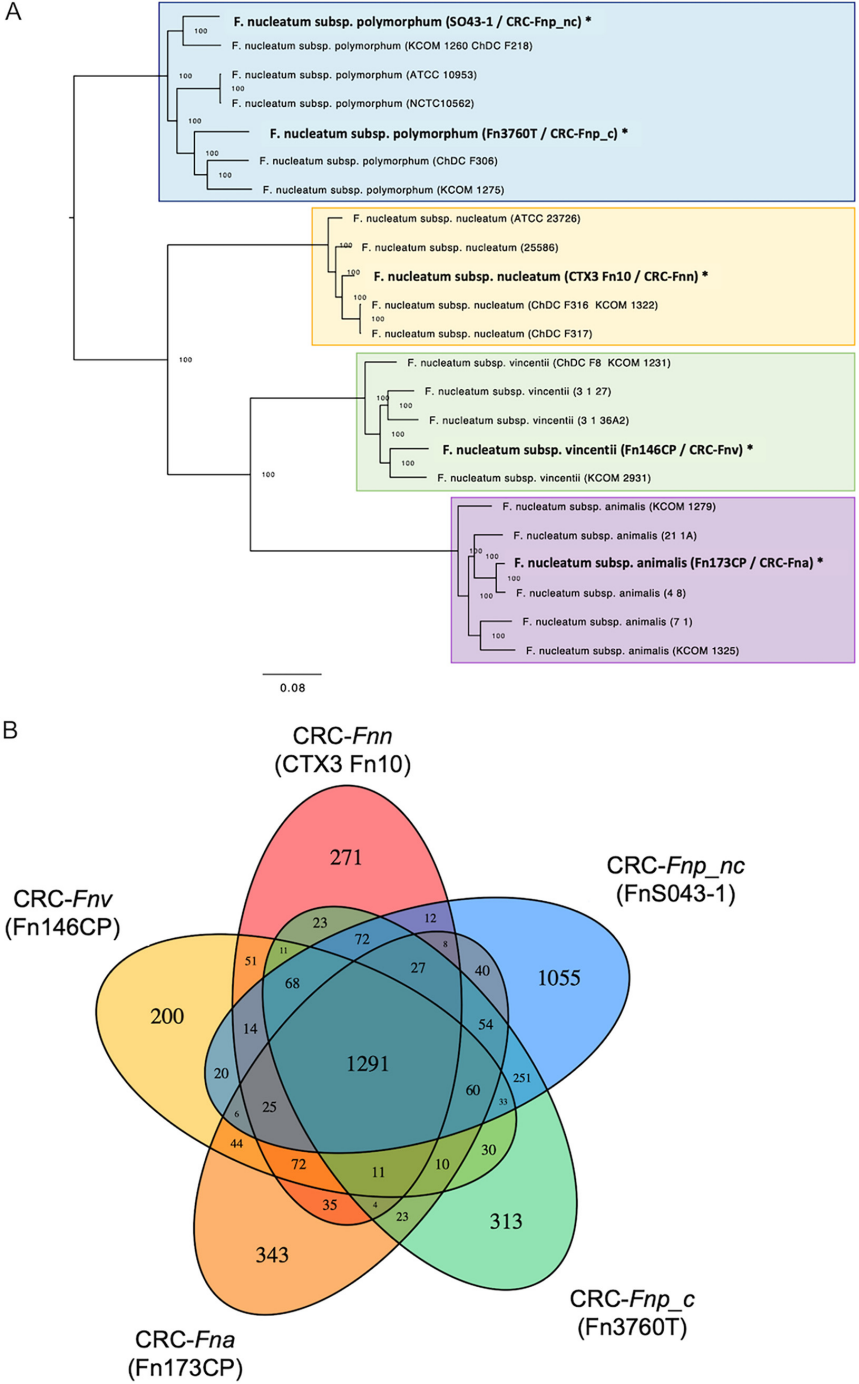
(A) Phylogenetic tree of 18 previously published whole-genome sequences of F. nucleatum strains aligned with 5 genomes newly sequenced for this study (in bold and marked with an asterisk). (B) Venn diagram depicting genomic comparison of the five sequenced CRC-derived F. nucleatum strains. Each strain is represented by a colored oval. The numbers represent the predicted protein coding genes unique to or shared by each strain, based on an 80% identity requirement. (*Fna* denotes F. nucleatum subsp. *animalis*, *Fnn* denotes F. nucleatum subsp. *nucleatum*, *Fnv* denotes F. nucleatum subsp. *vincentii*, *Fnp_c* denotes clumpy F. nucleatum subsp. *polymorphum*, and *Fnp*_*nc* denotes nonclumpy F. nucleatum subsp. *polymorphum*).

Given the considerable variation in *in vitro* growth characteristics, ability to colonize our murine models, and effects on inflammatory signaling, we performed whole-genome sequencing (WGS) of our CRC-derived F. nucleatum strains to begin to investigate potential reasons for these varied behaviors. Eighteen previously published whole-genome sequences were included in comparative analyses (see Table S5 at https://github.com/JessicaRQueen/Queen.Domingue.mBio2022). Each of our sequenced CRC strains aligned overall with other isolates from the same subspecies regardless of clinical source (Fig. 6A). Principal-coordinate analysis (PCoA) of average nucleotide identity revealed that all sequenced F. nucleatum strains clustered tightly according to subspecies; within a given subspecies, strain source did not account for observed genomic variation, which was relatively minor compared to variation between subspecies (Fig. S7). Among the CRC-derived F. nucleatum strains, we identified a core genome consisting of 1,291 protein-coding sequences (based on an 80% identity requirement) and between 200 and 1,055 protein-coding genes unique to each individual strain (Fig. 6B).

10.1128/mBio.02991-21.10 FIG S7 Average nucleotide identity matrix, converted to dissimilarity matrix, followed by principal coordinates analysis and PERMANOVA to evaluate factors associated with genomic variation. Download FIG S7, TIF file, 2.3 MB. Copyright © 2022 Queen et al. 2022 Queen et al. https://creativecommons.org/licenses/by/4.0/ This content is distributed under the terms of the Creative Commons Attribution 4.0 International license.

We next evaluated all 23 complete genome sequences for the presence of several key genes encoding proteins that have been implicated as important in CRC animal models, interactions with CRC cell lines, or aggregation with other bacteria in polymicrobial biofilms (Table S1). Highly conserved F. nucleatum genes *recA* and *nusG* were analyzed in parallel and confirmed to be present in all 23 F. nucleatum strains. All sequenced F. nucleatum strains possessed the gene for FadA, an adhesin that binds E-cadherin, promoting cell proliferation and inflammation in a mouse xenograft model (14). There were minimal single nucleotide polymorphisms (SNPs) in *fadA*, with all strains displaying >95% *fadA* sequence identity. There are three described homologues of FadA (31), and we found that the presence of FadA2 varied between strains. As previously reported (31), FadA3 was universally present, and several F. nucleatum strains had multiple copies.

10.1128/mBio.02991-21.2 TABLE S1 Presence or absence of F. nucleatum virulence and adhesion factor gene sequences (Aid1, CmpA, FadS, Fap2, FomA, RadD), with two control genes (RecA, 16S). Pos (green) denotes positive (≥50% of a reference gene; average identity, 94.8%). Ind (yellow) denotes indeterminate (<50% coverage; average identity, 83.8%). Neg (red) denotes negative (absence of alignment). CRC-derived strains listed in bold. (*Fna* denotes F. nucleatum subsp. *animalis*, *Fnn* denotes F. nucleatum subsp. *nucleatum*, *Fnv* denotes F. nucleatum subsp. *vincentii*, *Fnp_c* denotes clumpy F. nucleatum subsp. *polymorphum*, and *Fnp*_*nc* denotes nonclumpy F. nucleatum subsp. *polymorphum*). Download Table S1, PDF file, 0.02 MB. Copyright © 2022 Queen et al. 2022 Queen et al. https://creativecommons.org/licenses/by/4.0/ This content is distributed under the terms of the Creative Commons Attribution 4.0 International license.

We also assessed for the presence of Fap2, a surface protein and galactose-binding leptin critical for colonization in the orthotopic murine model (15). Many strains lacked a *fap2* gene with high sequence homology to the F. nucleatum 23726 reference strain studied in the mouse model in which Fap2 mediated impairment of antitumor immunity (15). CRC*-*F. nucleatum subsp. *animalis* and clumpy CRC*-*F. nucleatum subsp. *polymorphum* had sequences with only 67% alignment with the reference Fap2 and were more closely aligned with a sequence annotated as an autotransporter-associated N-terminal domain-containing protein. It is therefore unclear if these sequences encode highly divergent Fap2 proteins or alternate proteins with a conserved autotransporter domain. Both CRC-derived F. nucleatum subsp. *polymorphum* strains lacked the gene for RadD, an outer membrane protein that mediates binding to other bacterial species (33–35). However, this is not unique to the *polymorphum* subspecies, as RadD is also absent in one previously sequenced F. nucleatum subsp. *animalis* strain (Table S1). CmpA and Aid1 are two outer membrane proteins implicated in bacterial aggregation and biofilm formation (36, 37); whereas Aid1 was present in all sequenced F. nucleatum strains, presence of CmpA varied, with <30% BLASTN alignment coverage to the F. nucleatum 23726 reference sequence in many isolates, including both F. nucleatum subsp. *polymorphum* strains sequenced in this study. All F. nucleatum strains carried the gene for FomA, a voltage-dependent porin that acts as a TLR2 agonist and has been suggested as a possible self-adjuvanted antigen for F. nucleatum vaccination (38). Overall, analysis of these genes hypothesized to be clinically significant failed to demonstrate any distinct characteristics of CRC-derived strains compared to non-CRC strains (Table S1).

**FIG 7:**
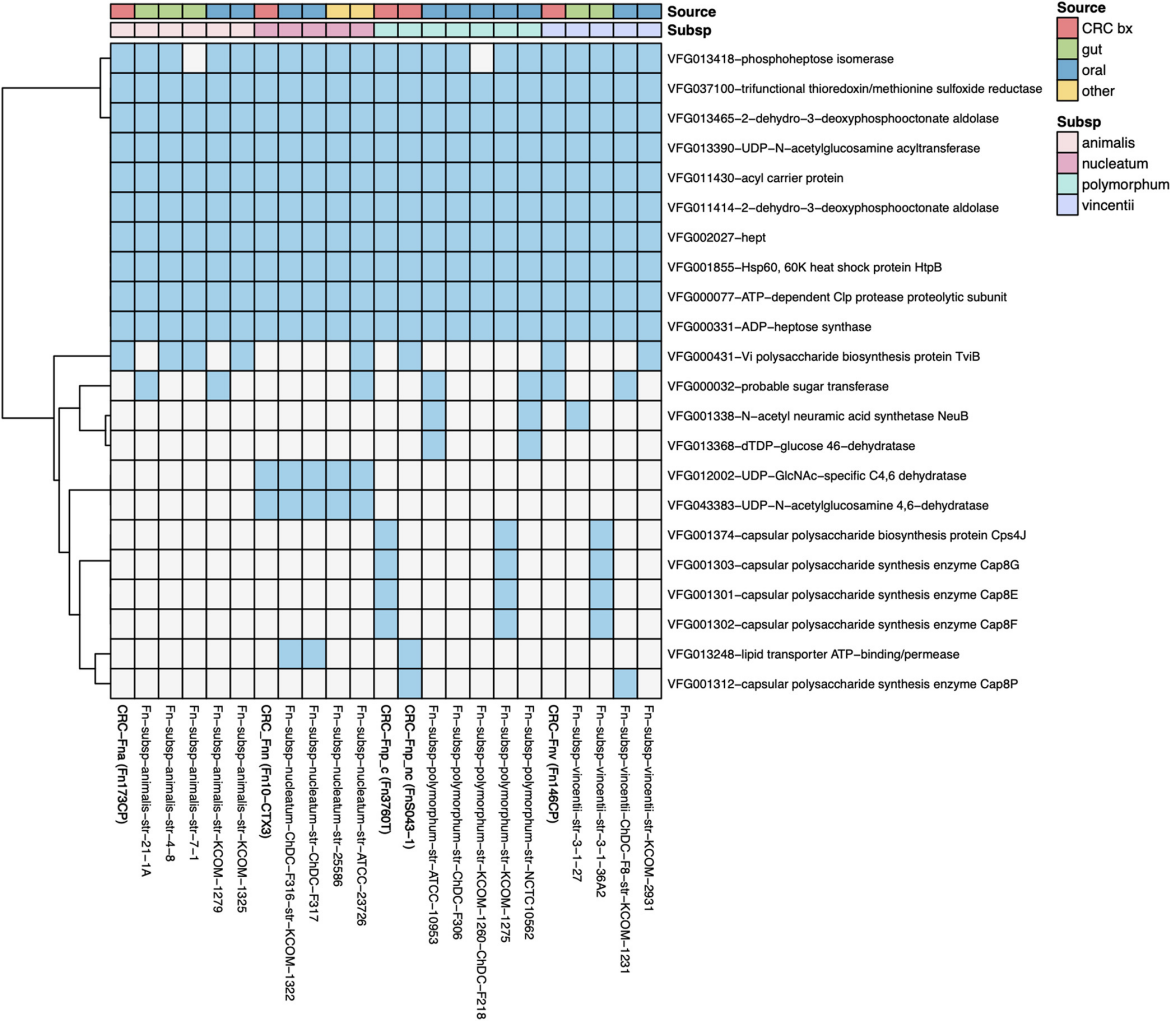
Heat map displaying virulence factors from the VFDB database observed in at least 2 of the 23 F. nucleatum isolates. Blue denotes presence, and white denotes absence. Analysis was performed on predicted protein sequences by applying BLASTP and requiring >50% amino acid identity with >85% coverage. Source of isolates (CRC biopsy specimen, gut, oral, or other) and subspecies are denoted in the color-coded legend.

There is little known about critical virulence factors expressed by F. nucleatum in the tumor microenvironment. Therefore, to identify potential additional gene products of clinical relevance, we performed a comparative pathogenomic analysis of F. nucleatum strains using the VFDB database of virulence factors expressed by pathogenic bacteria (Fig. 7) (39), which has previously been used to analyze F. nucleatum strains for their pathogenic potential (40). In our analysis, there were a number of gene products shared by all sequenced strains (see Table S3 at https://github.com/JessicaRQueen/Queen.Domingue.mBio2022). For example, all strains had the gene for ADP-heptose synthase, involved in generation of a lipopolysaccharide (LPS) biosynthesis pathway intermediate that has been shown to function as a pathogen-associated molecular pattern (PAMP) in multiple Gram-negative organisms (41). A number of other genes involved in LPS biosynthesis were shared by all strains. There were no virulence genes identified unique to the CRC-derived F. nucleatum genomes, with the exception of nonclumpy CRC-F. nucleatum subsp. *polymorphum* (F. nucleatum S043-1), which encoded 25 virulence genes not shared by any of the 22 other F. nucleatum strains. This strain had >75% sequence homology to several bacterial iota toxins, including Clostridioides difficile transferase A and B (CdtA and CdtB) and Clostridium perfringens iota toxin component lb, which ADP-ribosylates actin. This strain also possessed genes related to secretion, including general secretion pathway protein E, type IV pilus assembly protein PilB, and type VI secretion system AAA-positive (AAA^+^) family ATPase. Nonclumpy CRC-F. nucleatum subsp. *polymorphum* also had genes with homology to *Listeria* adhesion protein (Lap) and fibronectin-binding protein, which facilitate adherence to the intestinal epithelium. When we expanded our analysis to two additional virulence factor databases, Victors and PATRIC (42, 43), we identified numerous additional virulence genes unique to the nonclumpy CRC-F. nucleatum subsp. *polymorphum* strain (see Table S3 at https://github.com/JessicaRQueen/Queen.Domingue.mBio2022) and the CRC-F. nucleatum subsp. *vincentii* strain had a single unique virulence gene, asparagine synthetase AsnA. However, similar to the VFDB database, the Victors and PATRIC databases did not identify any gene signatures uniquely shared among the CRC-derived strains.

## DISCUSSION

The association of Fusobacterium nucleatum with human CRC has added to our growing understanding of the critical role of the microbiota in development and progression of colon tumors, with implications for early detection, prevention, and predicting responses to therapy. It remains an open question whether F. nucleatum functions in the tumor microenvironment as a tumor inducer, potentiator, or merely a colonizer. Although we have convincing data from murine models that individual bacteria can induce or enhance colon tumorigenesis (e.g., enterotoxigenic Bacteroides fragilis and colibactin-producing Escherichia coli) (44, 45), it is not yet known whether putative procarcinogenic bacteria, alone or in a consortium, promote and alter tumor formation in the human colonic microenvironment. Microbiota dysbiosis and resultant inflammation are hypothesized to drive carcinogenesis (46). In contrast, available data on the contributions of F. nucleatum to CRC pathogenesis from murine models is limited and of uncertain validity because a stable colonization model, which mimics the anticipated condition in humans, has not yet been reported. Daily gavage of an organism in mice may represent an antigen stimulation model leading to the low-level colon tumorigenesis reported to date. In prior studies, daily F. nucleatum inoculation was associated with NF-κB activation (13), which we speculate may have promoted the modest tumor induction reported through a nonspecific response to repeated exposure to LPS and other Gram-negative PAMPs.

Herein, we sought first to test potential conditions in SPF mice that are expected to yield stable colonization. However, despite repeated gavages of F. nucleatum, neither persistent colonization nor tumorigenesis was observed. One possibility for the difficulty of establishing a robust SPF murine model for F. nucleatum colon tumorigenesis is that mice are not a natural host of F. nucleatum, which may be a highly human-adapted organism. Thus, mouse colon epithelial and/or immune cells may lack critical receptors for F. nucleatum adhesion factors that are essential for persistent colonization. For example, Fap2 expressed by F. nucleatum binds to human, but not murine, TIGIT, an inhibitory receptor that suppresses antitumor immunity (47). Our data indicate that SPF mice have a high resistance to colonization despite reduction of the microbiota with broad-spectrum antibiotics and repeated F. nucleatum inoculations. Although we could successfully colonize GF mice after multiple inoculations, many were ultimately able to clear F. nucleatum, suggesting even the less developed GF mouse immune system can eliminate F. nucleatum from the murine colon. In addition, although we hypothesized that F. nucleatum would induce colon biofilms given its well-described role as a facilitator of polymicrobial biofilm formation and maturation in the oral mucosa, F. nucleatum biofilms were not observed in well-colonized GF WT mice.

Further, our studies in the GF *Apc^Min/+^* model of CRC suggest that colonization with F. nucleatum alone is insufficient to induce formation of colonic tumors and suggests that, unexpectedly, persistent F. nucleatum colonization may exert an antitumor effect. This lack of tumor induction by F. nucleatum was observed across all subspecies tested despite variable modest distal colon inflammatory gene expression not accompanied by histopathologic inflammation. These data suggest that if F. nucleatum subspecies induce colon tumorigenesis, this may require a specific consortium of cross-communicating or synergistic bacteria. Our data demonstrating a lack of tumor induction by F. nucleatum in the GF *Apc^MinΔ716/+^* mouse model are supported by an earlier study in which GF *Apc^MinΔ850/+^* mice were administered a weekly gavage of a human CRC-derived F. nucleatum isolate or a combination of 6 F. nucleatum isolates from subsp. *animalis*, *nucleatum*, and *vincentii* (28). In parallel studies, F. nucleatum was gavaged following administration of an intact SPF microbiota. After 20 weeks, mice in all groups were assessed, and no significant intestinal tumorigenesis was observed.

In contrast, our studies and others in human-derived CRC cell lines suggest that when human tumor cells are present, some strains of F. nucleatum can be potent inducers of proinflammatory cytokines that are known to promote tumor cell migration and invasion (30). Herein, we demonstrate that F. nucleatum strains, across subspecies, can promote inflammatory signaling in a human colon cancer cell line; however, the magnitude of inflammation varied between isolates. These data highlight that F. nucleatum strains isolated from colonic tumors and other clinical specimens differ in their ability to promote inflammation *in vitro* and possibly in the human host. Given the weak F. nucleatum phenotypes in mouse models, further studies of humans with CRC with and without F. nucleatum colonization are needed to understand the contribution of F. nucleatum to human colon carcinogenesis.

Although sequencing analysis has demonstrated an enrichment of F. nucleatum in the tumor-associated microbiota of a proportion of CRC patients (7, 13), we lack a clear understanding of which subspecies are most relevant. F. nucleatum subspecies can be differentiated biochemically and genomically (2, 48). Based on average nucleotide identity or genome-to-genome analysis of whole-genome sequences, it has recently been suggested that there is sufficient genetic heterogeneity between the subspecies that they should be reclassified as separate species (49). Analysis of cancer-associated microbiota that relies on partial sequencing of the 16S rRNA gene lacks the resolution to identify the F. nucleatum subspecies in clinical samples. Therefore, sequencing paired with culture methods to isolate and further characterize specific F. nucleatum strains is needed. A novel PCR-based method for distinguishing the F. nucleatum subsp. was recently reported (50); validation of this promising technique in geographically diverse patient samples will be helpful given our findings of significant sequence variation in our nonclumpy CRC-F. nucleatum subsp. *polymorphum* strain collected in Malaysia compared to clinical isolates from Western Europe and the United States.

The limited number of fully sequenced F. nucleatum genomes indicates a high degree of genetic variation between strains. Our whole-genome sequencing analysis supports this, with CRC-derived strains clustering with other isolates from the same subspecies, but with significant variability in genes encoding proteins that have been implicated in existing CRC animal models. In particular, we observed significant variation in Fap2, where it is either absent or highly divergent in several F. nucleatum isolates. Furthermore, we identified several virulence genes unique to just the nonclumpy CRC-F. nucleatum subsp. *polymorphum* strain. It is possible that the high degree of divergence observed in this strain reflects geographic diversity, as this was the only CRC-derived strain in our cohort isolated in Asia. Previous genomic analysis of the F. nucleatum subsp. *polymorphum* strain ATCC 10953 revealed that 25% of the protein-coding genes were unique to that strain, with evidence of horizontal gene transfer with *Firmicutes*, particularly *Clostridia* (51). It is unclear whether this genetic tractability is unique to F. nucleatum subsp. *polymorphum* or if this is a strain-specific property. Further studies will be necessary to assess expression and function of F. nucleatum genes of interest in various experimental conditions.

In conclusion, we have demonstrated that stable colonization of GF *Apc^Min/+^* mice with CRC-derived F. nucleatum may modestly modulate host immune responses but does not promote colonic tumor formation in a monocolonized gnotobiotic mouse model. Behavior of F. nucleatum isolates in both *in vitro* and *in vivo* assays differed at the subspecies and strain levels, but there was no clear genomic distinction between tumor-associated F. nucleatum isolates and other clinical strains. We hypothesize that F. nucleatum may act in concert with other bacteria to activate essential carcinogenic signaling and promote formation or progression of colon tumors. However, differences in human and murine immune signaling may account for the lack of tumor promotion in mouse models.

Future studies on the role of F. nucleatum in colonic tumorigenesis will benefit from development of robust, persistently colonized animal models of CRC that examine F. nucleatum in association with other members of the colon and oral microbiota that are enriched in colonic tumors. Examination of subspecies- and strain-specific phenotypes in robust F. nucleatum models, with particular emphasis on investigation of CRC-derived clinical isolates, will be critical to further define the role of F. nucleatum in colon tumorigenesis and to delineate mechanisms of pathogenesis.

## MATERIALS AND METHODS

### Culturing and inoculum preparation.

F. nucleatum strains used in this study (Table 1) were either previously isolated from CRC biopsy tissue or other clinical sites and obtained from the sources listed in Table 1 or were isolated for this study from human CRC biopsy specimens using selective culture media (*Fusobacterium* selective agar; Anaerobe Systems) and identified by colony PCR with *Fusobacterium*-specific 16S primers (forward, 5′-GGATTTATTGGGCGTAAAG-3′C; reverse, 5′-GGCATTCCTACAAATATCTACGAA-3′) (52), and the species and subspecies were confirmed by Sanger sequencing of the 16S rRNA gene. F. nucleatum strains were cultured in brain heart infusion (BHI) media supplemented with hemin (10 μg/mL) and vitamin K (5 μg/mL) under anaerobic conditions (75% N_2_, 5% H_2_, 20% CO_2_) at 37°C for 48 to 72 h in static culture (to an optical density at 600 nm [OD_600_] of ∼1.5). Bacterial cultures were centrifuged and pellets resuspended in phosphate-buffered saline (PBS) under anaerobic conditions prior to animal inoculation.

### Murine models.

Wild-type C57BL/6J (WT; Jackson Laboratories) and multiple intestinal neoplasia mice (*Apc^MinΔ716/+^*, more permissive to intestinal tumorigenesis than *Apc^MinΔ850/+^* mice [53], termed *Apc^Min/+^* here; from David Huso, Johns Hopkins University) were housed in either the specific-pathogen-free (SPF) or germfree (GF) barrier facilities, as indicated. Six- to 10-week-old male and female mice were used for all experiments. Mice were maintained on a 12-h light/dark cycle and fed standard rodent chow *ad libitum*. For SPF experiments, we used two approaches. In the first, WT mice were treated with cefoxitin (5 mg/mL) *ad libitum* in drinking water for 48 h and then supplied with normal water for 36 h prior to the first inoculation; this approach was previously shown to temporarily eliminate detectable gut bacteria (54). Mice were maintained on gentamicin (35 μg/mL) *ad libitum* in drinking water for the duration of the experiment to further foster a dysbiotic microbiota potentially permissive to *Fusobacterium* spp. colonization. Antibiotic-treated SPF WT mice were inoculated via orogastric administration with 200 μL of F. nucleatum inoculum (equivalent to ∼10^9^ genome copies) once weekly for 4 weeks. In our second approach, SPF *Apc^Min/+^* mice were treated with streptomycin (5 mg/mL) and clindamycin (0.1 mg/mL) *ad libitum* in drinking water for 5 days prior to the first inoculation with normal water provided for the experiment duration. These mice were treated with an intensified gavage scheme of orogastric inoculum gavage three times in the first week, followed by weekly gavage for 10 weeks.

GF WT and *Apc^Min/+^* mice were inoculated in a laminar flow hood via orogastric gavage with 200 μL of F. nucleatum inoculum once weekly for 4 weeks, except for 2-week-long experiments where F. nucleatum was administered once. Individual GF cages (Allentown, Inc., NJ) were used for each experimental group to prevent cross-contamination of F. nucleatum strains. Mice were euthanized at the indicated time points and tissues harvested for further analyses. Fecal F. nucleatum colonization was assessed via reverse transcription-quantitative PCR (qRT-PCR) analysis of the *Fusobacterium* 16S rRNA gene. Distal colon gene expression was assessed by qRT-PCR using TaqMan gene expression assays, and each target gene was normalized to murine GAPDH (glyceraldehyde-3-phosphate dehydrogenase) and/or GusB. Downstream processing of mouse stools and tissues is further described in Text S1 in the supplemental material. The Johns Hopkins University Animal Care and Use Committee approved all experimental protocols, and all studies were in accordance with Animal Research: Reporting of *In Vivo* Experiments (ARRIVE) guidelines.

10.1128/mBio.02991-21.1 TEXT S1 Supplemental methods. Download Text S1, DOCX file, 0.03 MB. Copyright © 2022 Queen et al. 2022 Queen et al. https://creativecommons.org/licenses/by/4.0/ This content is distributed under the terms of the Creative Commons Attribution 4.0 International license.

### *In vitro* cytokine release assay.

As previously described (30), HCT116 (ATCC CCL-247) cells were grown on tissue culture-treated plates and flasks in McCoy’s 5A medium supplemented with 10% fetal bovine serum (FBS), penicillin, and streptomycin. HCT116 cells were seeded to confluence in 24-well plates (2 × 10^5^ cells per well at 100% confluence), and F. nucleatum subspecies were added at a multiplicity of infection (MOI) of 50:1 followed by incubation at 37°C and 5% CO_2_ for 4 h. Medium from individual wells was sterile filtered using a 0.2-μm filter (MilliporeSigma) and diluted to concentrations within the range of the R&D Systems DuoSet enzyme-linked immunosorbent assay (ELISA) to analyze human IL-8 and CXCL1 concentrations.

### WGS and comparative genome analysis.

WGS was done at the PennCHOP Microbiome Center (University of Pennsylvania) using Illumina MiSeq technology as described in Text S1. WGS data sets were trimmed for quality using FASTP v0.20.0 (55), assembled using SPAdes v3.14.1 (56), and annotated for gene content using the DFAST pipeline v1.2.6 (57) and PROKKA v1.14.5 (58), followed by MetaCyc pathway analysis with MinPath (59). Genome sequences of the 5 newly sequenced CRC-derived F. nucleatum strains were compared to 18 previously sequenced complete genomes, all of which were non-CRC-derived isolates and were inclusive of strains F. nucleatum 23726 and F. nucleatum EAVG_002 described in Table 1. Whole-genome alignment of sequenced and reference *Fusobacterium* isolates was performed using MUGSY v1r2.3 (60). Core alignment blocks (≥1,000 bp) covering all considered strains were concatenated into a larger alignment (totaling 1,362,030 bp). This alignment was narrowed to positions containing SNPs (*n* = 240,340) and submitted to FastTree v1 (61) for phylogenetic tree construction. To study whole-genome variation between and within subspecies and by source, the concatenated whole-genome alignment was used to calculate average nucleotide identity (ANI) between all strains counting SNP-based mismatches that were observed ≥100 bp from the end of each original core alignment block. ANI measures were then converted to a dissimilarity matrix, followed by principal-coordinate analysis (PCoA) and permutational multivariate analysis of variance (PERMANOVA) using the Vegan R package v2.5.6. To evaluate shared and unique gene content, coding sequences from each of the five F. nucleatum isolates were clustered using USEARCH v11.0.667 (62) with an 80% identity threshold and visualization of overlapping membership per cluster by VennDiagram in R v3.5.3. Virulence factor searches were performed on predicted protein sequences of 23 F. nucleatum strains using the PATRIC (42), Victors (43), and VFDB (39) databases by applying BLASTP and requiring >50% amino acid identity with >85% coverage. VFDB categories with at least two positive calls based on identity and coverage criteria were analyzed as binary data for heatmap generation using the pheatmap R package (v1.0.12) and specifying the Manhattan distance to cluster virulence factor categories. Nondefault parameter settings for all computational analyses are available in Table S4 at https://github.com/JessicaRQueen/Queen.Domingue.mBio2022. Name, accession number, and clinical source for the 18 previously sequenced strains and the 5 strains sequenced for this study are listed in Table S5 at https://github.com/JessicaRQueen/Queen.Domingue.mBio2022.

### Statistical analysis.

For colonization analyses, murine groups were analyzed using a Mann-Whitney (nonparametric) test. For comparative analysis of gene expression levels and tumorigenesis, we performed a nonparametric Kruskal-Wallis one-way ANOVA; significant pairwise comparisons were subsequently analyzed by Mann-Whitney test. Linear regression was performed for correlation of gene expression and F. nucleatum copy number (analyzed by qRT-PCR as described in Text S1 at https://github.com/JessicaRQueen/Queen.Domingue.mBio2022). Fisher’s exact test was performed for comparison of tumorigenesis in F. nucleatum-colonized versus cleared mice. Results from *in vitro* assays were analyzed by parametric one-way ANOVA with Tukey’s test for multiple comparisons. Differences with a *P* value of <0.05 were considered significant.

### Data availability.

Whole-genome sequencing data have been submitted to NCBI under NCBI BioProject accession ID PRJNA755318. Accession numbers for the 5 strains sequenced for this work as are follows: for Fn146CP, SAMN20819806; for Fn173CP, SAMN20819807; for CTX3Fn10, SAMN20819805; for Fn3760T, SAMN20819808; and for FnS043-1, SAMN20819809.
