# Supplementary figures and images for: Comparative Analysis of Colon Cancer-Derived Fusobacterium nucleatum Subspecies: Inflammation and Colon Tumorigenesis in Murine Models
Source: mBio. 2022 Feb 8;13(1):e02991-21. doi: 10.1128/mbio.02991-21 (PMC8822350; doi:10.1128/mbio.02991-21)

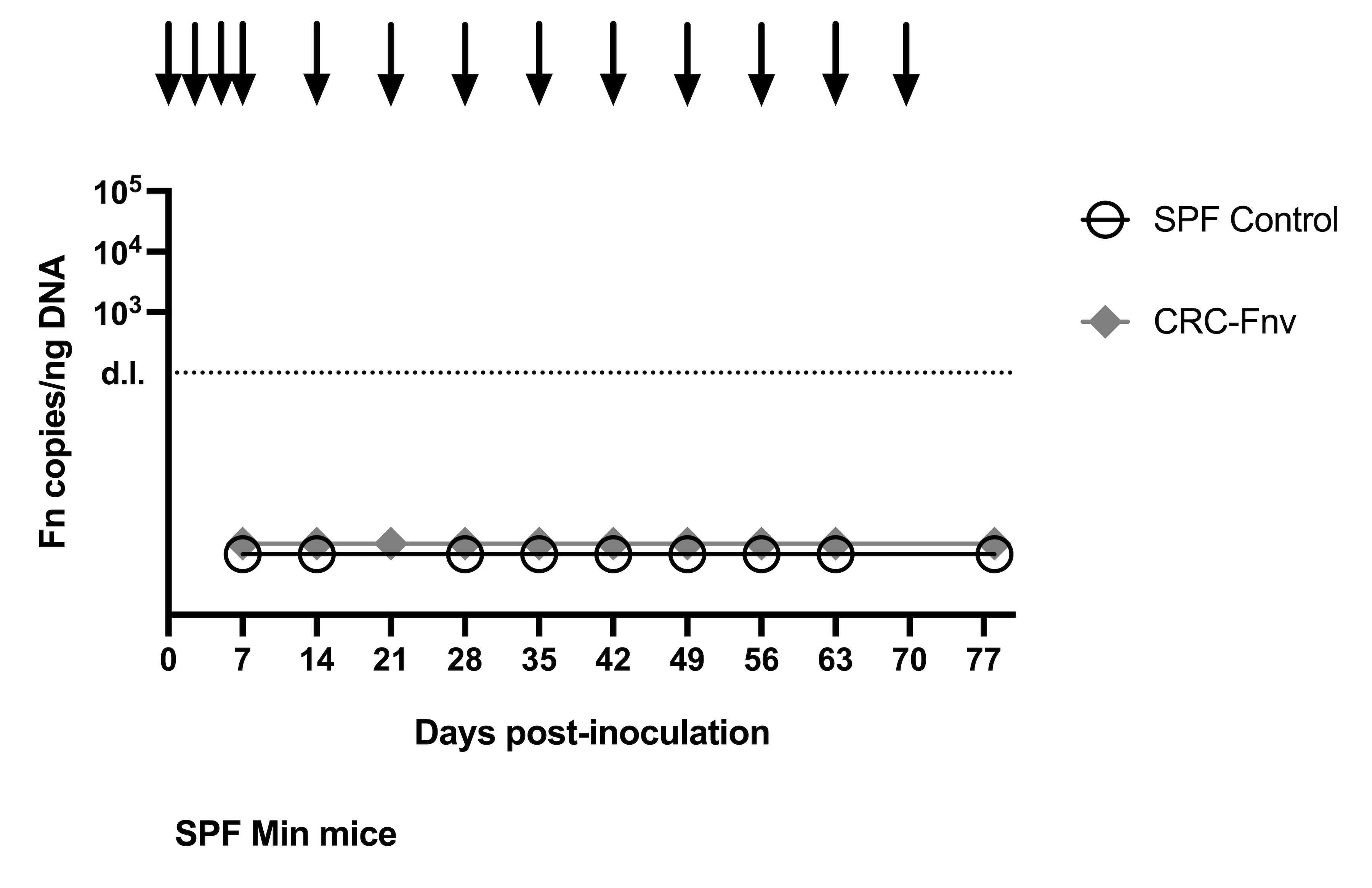

Supplement: FIG S1 [file mbio.02991-21-sf001.tif]

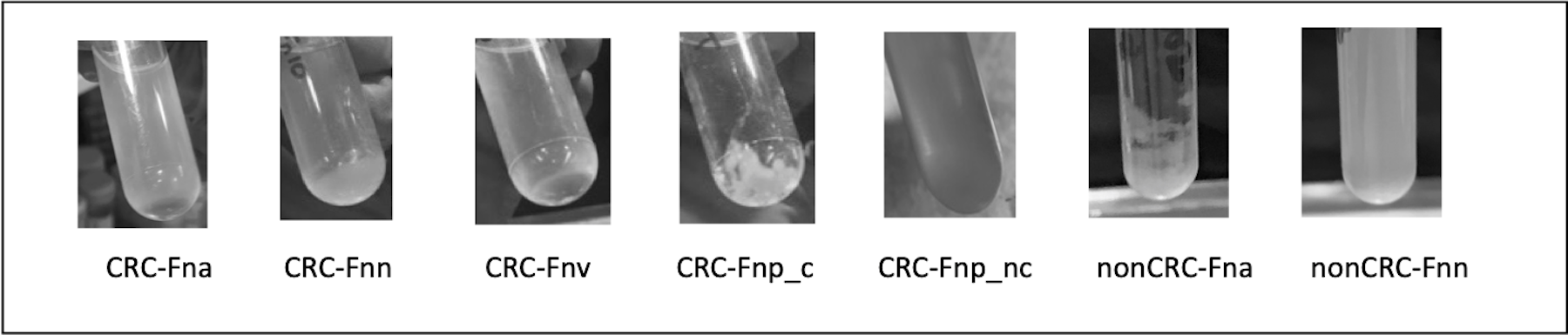

Supplement: FIG S2 [file mbio.02991-21-sf002.tif]

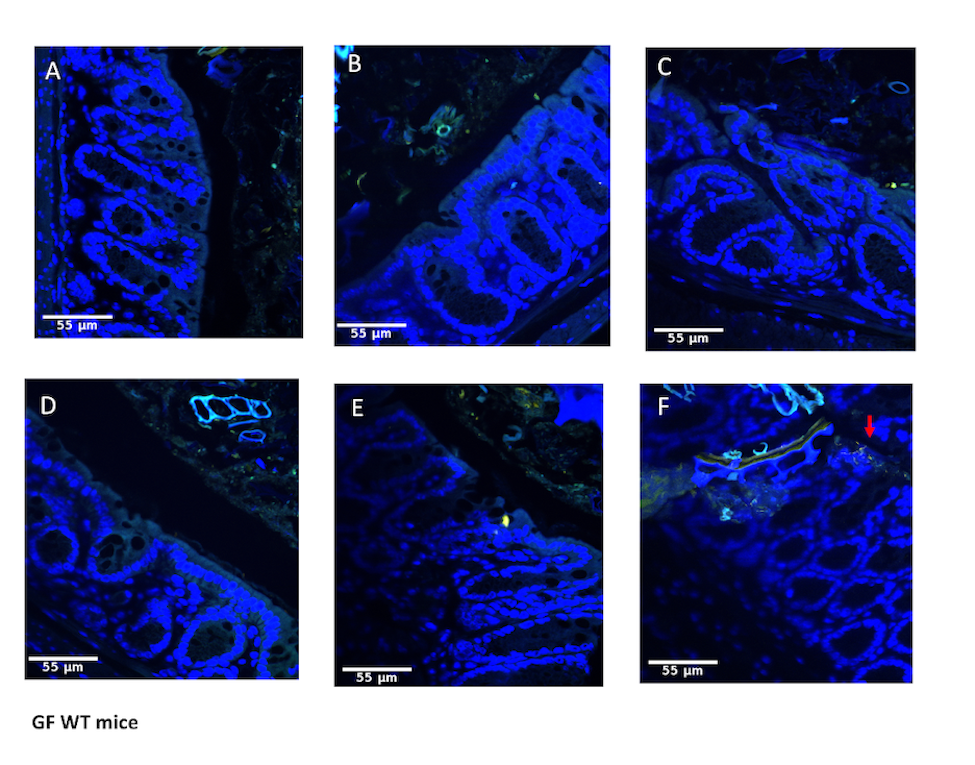

Supplement: FIG S3 [file mbio.02991-21-sf003.tif]

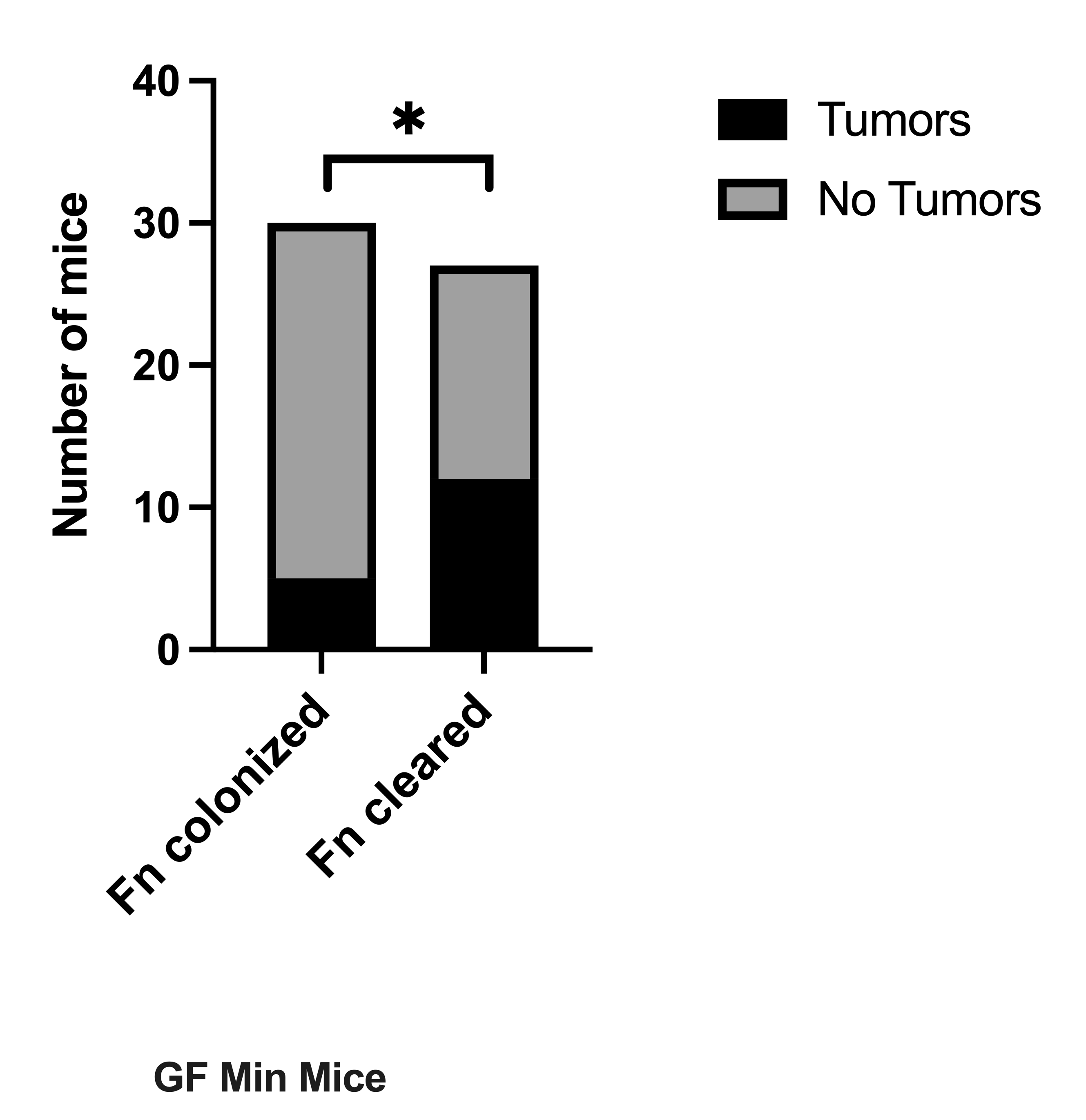

Supplement: FIG S4 [file mbio.02991-21-sf004.tif]

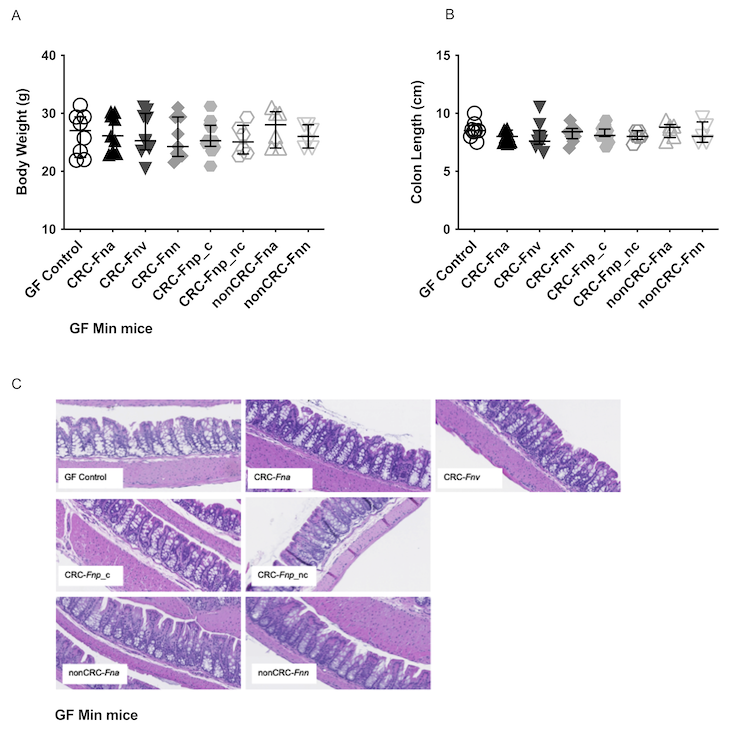

Supplement: FIG S5 [file mbio.02991-21-sf005.tif]

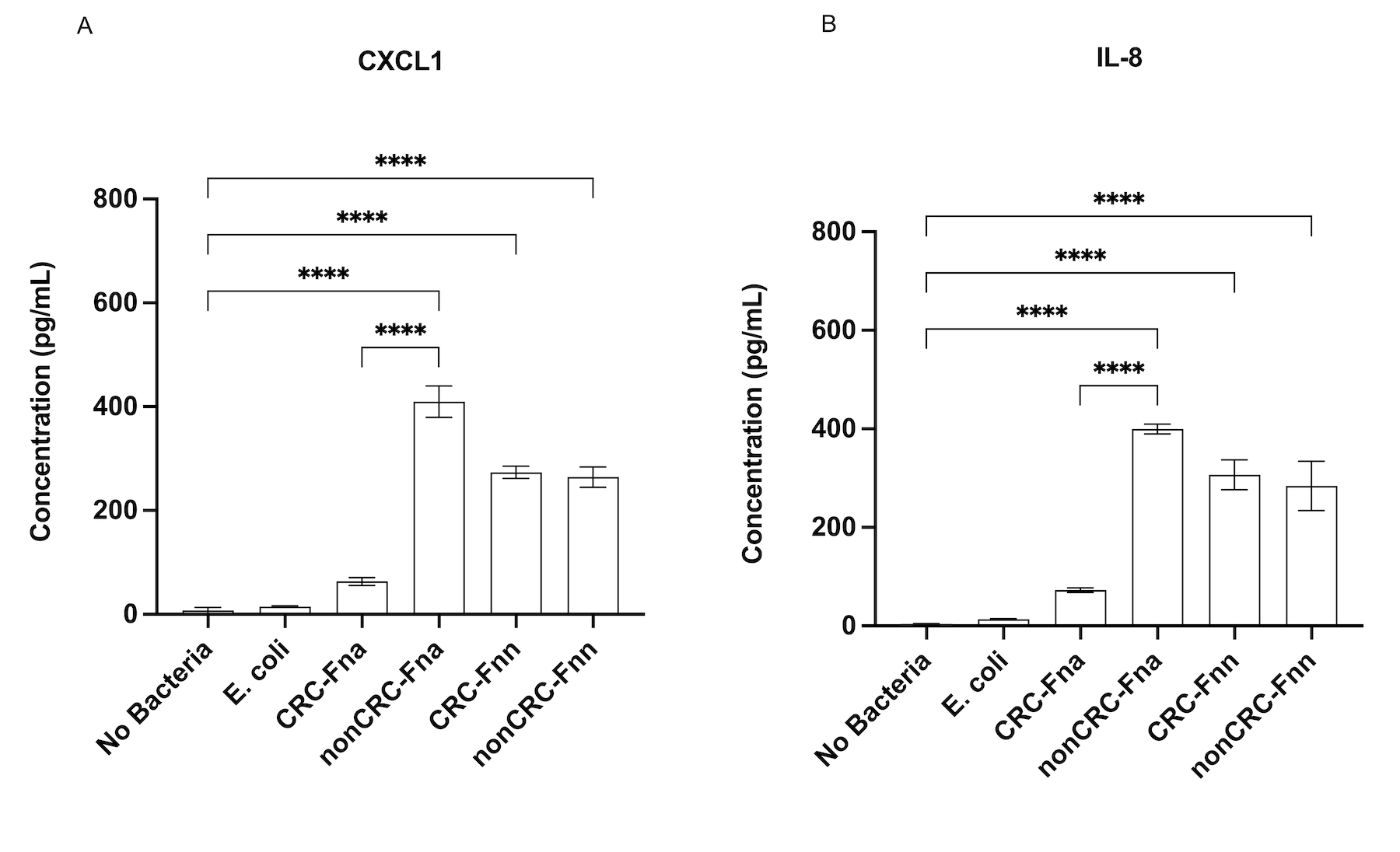

Supplement: FIG S6 [file mbio.02991-21-sf006.tif]

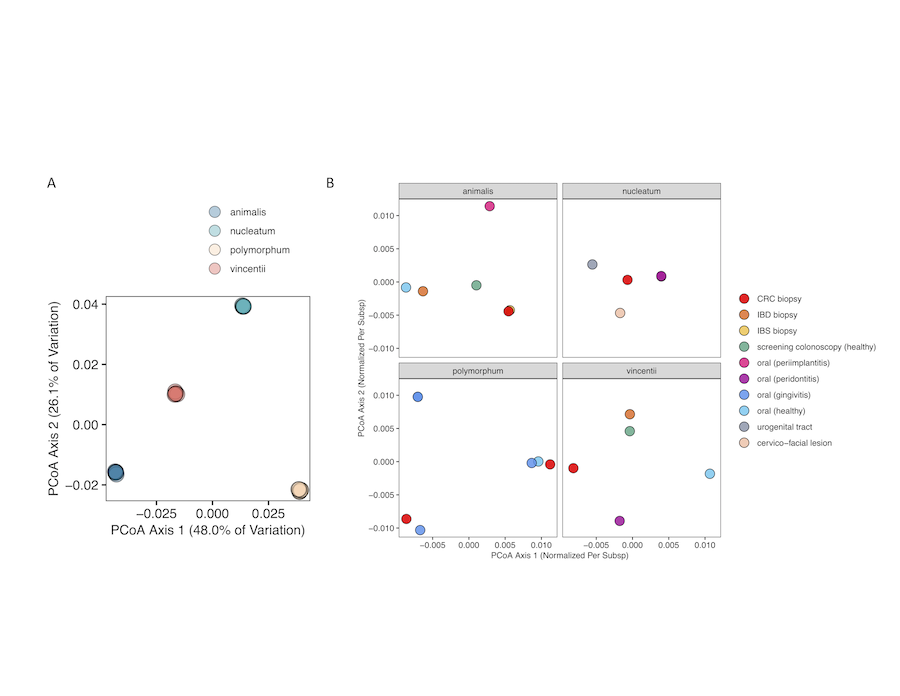

Supplement: FIG S7 [file mbio.02991-21-sf007.tif]
