# Supplementary material for: Comparative Analysis of Colon Cancer-Derived Fusobacterium nucleatum Subspecies: Inflammation and Colon Tumorigenesis in Murine Models
Source: mBio. 2022 Feb 8;13(1):e02991-21. doi: 10.1128/mbio.02991-21 (PMC8822350; doi:10.1128/mbio.02991-21)
Supplement: TABLE S2 [file mbio.02991-21-st002.pdf]

Taqman Assay ID	Gene Symbol	Gene Name	GF Control	CRC-Fna		CRC-Fnv		CRC-Fnp_c		CRC-Fnp_nc		nonCRC-Fna		nonCRC-Fnn	
			Median 2^-DDCt	Median 2^-DDCt	p-value	Median 2^-DDCt	p-value	Median 2^-DDCt	p-value	Median 2^-DDCt	p-value	Median 2^-DDCt	p-value	Median 2^-DDCt	p-value
Mm01179194_m1	Cd3e	CD3 antigen, epsilon polypeptide	0.8230	**1.9421**	**0.0303**	3.4368	0.4762	2.4551	0.0952	0.8757	0.9143	0.5705	0.0952	0.7996	0.5476
Mm00442754_m1	Cd4	CD4 antigen	1.0063	2.2855	0.2468	2.0742	0.4762	2.3523	0.1667	0.6818	0.3524	0.4067	0.0952	**0.3128**	**0.0476**
Mm01182107_g1	Cd8a	CD8 antigen, alpha chain	0.7445	1.5568	0.0823	4.0686	0.7619	1.3837	0.2619	0.6751	0.4762	0.4458	0.1667	**0.5125**	**0.0476**
Mm00491152_m1	Cd33	CD33 antigen	1.0177	1.4162	0.0519	1.5338	0.1143	1.4910	0.2619	1.1442	0.6095	0.6400	0.1667	**0.4703**	**0.0476**
Mm01168134_m1	Ifng	interferon gamma	1.2507	1.0088	0.0823	1.7490	0.6095	0.7293	0.5476	0.7243	0.3524	0.5301	0.2619	0.7643	0.9048
Mm00439618_m1	Il17a	interleukin 17A	1.6370	7.5656	0.0519	10.2217	0.2571	7.3835	0.2619	6.1168	0.0095	0.8223	0.9048	0.9928	0.9048
Mm00443258_m1	Tnf	tumor necrosis factor	0.7542	2.0984	0.0519	3.8083	0.1143	1.6203	0.3810	0.6187	0.3524	**0.4537**	**0.0238**	0.5491	0.2619
Mm01226722_g1	Il22	interleukin 22	0.5905	4.0359	0.2468	0.2658	0.3524	0.2059	0.0952	1.3835	0.9143	0.0318	0.2619	1.7751	0.9048
Mm00446190_m1	Il6	interleukin 6	0.9152	2.3580	0.1775	2.0432	0.0667	1.8304	0.1667	**0.5707**	**0.0095**	0.5740	0.0238	**0.4939**	**0.0238**
Mm01288386_m1	Il10	interleukin 10	1.0088	1.4367	0.0519	2.7691	0.3524	0.8212	0.9048	0.8282	0.9143	0.9406	0.7143	0.5786	0.2619
Mm00434228_m1	Il1b	interleukin 1 beta	1.2860	1.3316	0.7922	1.0093	>0.9999	1.0212	0.5476	0.4133	0.0667	**0.2526**	**0.0476**	0.3463	0.1667
Mm00518984_m1	Il23a	interleukin 23, alpha subunit p19	1.2176	1.2688	0.2468	1.4392	0.6095	1.8551	0.1667	0.5753	0.1143	0.6842	0.3810	**0.3746**	**0.0238**
Mm00434169_m1	Il12a	interleukin 12a	0.8946	1.1749	0.2468	2.5205	0.7619	1.3065	0.5476	0.7544	0.7619	0.2800	0.1667	**0.2631**	**0.0238**
Mm00505403_m1	Il33	interleukin 33	1.0396	1.8047	0.3290	1.7600	**0.0381**	1.4368	0.0952	**0.5928**	**0.0381**	**0.4514**	**0.0238**	**0.3650**	**0.0238**
Mm00499822_m1	Il25	interleukin 25	1.0065	2.2492	0.5368	1.6891	0.2571	1.2097	>0.9999	0.9515	0.9143	0.5551	0.2619	0.4854	0.1667
Mm04207460_m1	Cxcl1	chemokine (C-X-C motif) ligand 1	0.9880	1.0458	0.0519	2.0145	0.9143	1.1441	0.9048	1.0128	>0.9999	0.8237	0.3810	1.9727	0.5476
Mm00436450_m1	Cxcl2	chemokine (C-X-C motif) ligand 2	0.9608	1.7079	0.0519	1.8420	0.1143	1.3627	0.3810	0.6301	0.2571	**0.4122**	**0.0238**	1.1646	>0.9999
Mm00434946_m1	Cxcl9	chemokine (C-X-C motif) ligand 9	1.1709	0.7002	0.2468	2.4060	0.9143	0.8548	0.9048	0.8753	>0.9999	0.2401	0.0952	0.2871	0.3810
Mm00475988_m1	Arg1	arginase, liver	1.0644	2.2673	0.1255	**2.6974**	**0.0190**	**2.9429**	**0.0476**	0.6112	0.1714	0.6647	0.2619	0.5440	0.0952
Mm00492590_m1	Ido1	indoleamine 2,3-dioxygenase 1	1.0176	1.0889	0.1775	2.1097	0.7619	0.9180	>0.9999	1.0519	0.7619	**0.4083**	**0.0238**	1.2939	0.7143
Mm03048248_m1	Cd274	CD274 antigen	1.0348	2.1192	0.1255	**2.1711**	**0.0381**	1.7904	0.1667	0.9224	0.3524	0.7909	0.5476	0.5852	0.5476
Mm01261022_m1	Rorc	RAR-related orphan receptor gamma	1.1857	1.6062	0.0823	1.6002	0.2571	1.3106	0.3810	**0.4145**	**0.0381**	0.5356	0.0952	**0.3550**	**0.0238**
Mm00450960_m1	Tbx21	T-box 21	0.8908	1.5865	0.4286	2.5513	0.4762	1.0343	0.7143	0.5584	0.1143	**0.4359**	**0.0238**	**0.3865**	**0.0476**
Mm00484683_m1	Gata3	GATA binding protein 3	1.1362	2.1470	0.0519	2.7862	0.1143	1.8443	0.3810	**0.4024**	**0.0381**	**0.3856**	**0.0238**	**0.3062**	**0.0238**
Mm00475162_m1	Foxp3	forkhead box P3	0.7760	2.1777	0.0519	4.3339	0.1143	2.8888	0.0952	0.9650	0.4762	0.3937	0.1667	0.3570	0.2619
Mm00434214_m1	Il17ra	interleukin 17 receptor A	1.2921	1.6407	0.1255	1.9773	0.1714	1.4454	0.3810	0.5146	0.0667	**0.3892**	**0.0476**	**0.3586**	**0.0238**
Mm00656927_g1	Saa1	serum amyloid A 1	0.8448	0.6455	0.4286	0.7247	0.2571	0.7590	0.3810	1.5345	0.2571	1.2168	0.7143	1.0552	0.7143
Mm00496696_g1	S100a8	S100 calcium binding protein A8 (calgranulin A)	1.1030	**1.0805**	**0.0303**	1.9850	0.9143	1.4515	0.3810	0.5894	0.1714	0.7237	0.9048	0.7091	0.2619
Mm00656925_m1	S100a9	S100 calcium binding protein A9 (calgranulin B)	1.6489	0.2927	0.4286	2.1177	0.6095	1.6195	0.9048	1.0736	0.4762	1.3931	0.9048	1.2681	0.9048
Mm03294838_g1	COX2	cytochrome c oxidase subunit II	0.5581	0.5559	0.4286	0.4764	0.7619	0.4697	0.3810	2.7556	0.3524	2.7208	0.3810	2.6132	0.5476
Mm01192943_m1	Il22ra1	interleukin 22 receptor, alpha 1	1.2812	**1.7519**	**0.0173**	1.7881	0.1714	1.3684	0.7143	0.5321	0.2571	0.4778	0.0952	**0.4478**	**0.0238**
Mm00432403_m1	Cd36	CD36 antigen	11.5013	13.2098	0.9307	18.5755	0.7619	16.3530	0.7143	6.7479	0.1714	7.6892	0.3810	5.2139	0.2619
Mm00463327_m1	Il1f9	interleukin 1 family, member 9	3.8584	6.5634	0.1255	7.5870	0.3524	**11.7625**	**0.0476**	2.2243	0.4762	3.5406	0.9048	1.8510	0.5476
Mm01329359_m1	Mrc1	mannose receptor, C type 1	1.0806	1.2952	0.3290	1.5779	0.1143	1.4477	0.2619	0.6651	0.0667	0.6531	0.0952	0.4642	0.0476
Mm01276696_m1	Muc2	mucin 2	0.9893	0.9161	0.1255	0.7819	0.9143	0.8513	0.2619	1.2244	0.4762	1.0239	0.9048	0.8547	0.0952
Mm00441127_m1	Reg3g	regenerating islet-derived 3 gamma	1.2784	1.3104	0.5368	1.6605	0.6095	1.2018	0.5476	0.3172	0.0667	0.2894	0.0952	**0.2234**	**0.0476**
Mm00657074_m1	Defb2	defensin beta 2	1.6580	1.9346	>0.9999	1.2791	0.3524	1.4203	0.9048	0.0939	0.0095	0.2194	0.5476	**0.0925**	**0.0238**
Mm00484518_s1	Fut1	fucosyltransferase 1	0.9259	1.8193	0.1775	2.1948	0.1143	1.6265	0.1667	0.9380	>0.9999	0.8458	0.7143	0.6156	0.1667
Mm00487804_m1	Myc	myelocytomatosis oncogene	1.0090	**1.6851**	**0.0043**	**2.3979**	**0.0190**	**1.8830**	**0.0476**	0.9249	0.6095	**0.6632**	**0.0238**	**0.6459**	**0.0238**
Mm01247357_m1	Cdh1	cadherin 1	1.1427	1.1114	0.9307	1.1595	>0.9999	1.2002	0.7143	1.0037	0.7619	0.7158	0.2619	**0.5175**	**0.0238**
Mm04204476_m1	Ceacam1	carcinoembryonic antigen-related cell adhesion	0.9916	0.9056	0.4286	0.8862	0.4762	0.8925	0.5476	0.9305	0.6095	0.8386	0.7143	**0.6986**	**0.0238**
Mm01208059_m1	Nos1	nitric oxide synthase 1, neuronal	0.9195	1.6594	0.6623	1.1369	0.2571	1.1787	>0.9999	0.6379	0.3524	0.7301	0.2619	0.4677	0.0952
Mm00549170_m1	Nox1	NADPH oxidase 1	1.1467	0.5763	0.2468	0.8723	0.2571	0.7177	0.7143	1.4104	0.3524	1.1463	0.9048	0.9288	0.7143
Mm01287743_m1	Cybb	cytochrome b-245, beta polypeptide	1.0728	1.6675	0.0519	2.4221	0.1714	1.8247	0.2619	0.8482	0.4762	0.4405	0.0952	**0.3876**	**0.0238**
Mm01298424_m1	Mpo	myeloperoxidase	2.1417	14.0747	>0.9999	0.1584	0.7619	0.1227	0.9048	13.6023	0.4762	14.8908	0.1667	0.0177	0.1667

Normalized to Housekeeping Genes:

Mm99999915_g1	Gapdh	glyceraldehyde-3-phosphate dehydrogenase
Mm01197698_m1	Gusb	glucuronidase, beta
