# Supplementary material for: Comparative Analysis of Colon Cancer-Derived Fusobacterium nucleatum Subspecies: Inflammation and Colon Tumorigenesis in Murine Models
Source: mBio. 2022 Feb 8;13(1):e02991-21. doi: 10.1128/mbio.02991-21 (PMC8822350; doi:10.1128/mbio.02991-21)
Supplement: TEXT S1 [file mbio.02991-21-s0001.docx]

**Text S1. Supplemental Methods**

Colonization Analysis by Fecal DNA

Mouse fecal pellets were collected and stored at -20°C until processing. Fecal DNA was extracted using the Zymo Quick-DNA Fecal/Soil Microbe 96 kit (D6011) per manufacturer’s instructions. *Fn* colonization was assessed via qPCR analysis of Fusobacterium 16S rRNA gene with specific primers (F: 5’-GGATTTATTGGGCGTAAAG-3’C, R: 5’-GGCATTCCTACAAATATCTACGAA-3’) (1) and probe (5'HEX-TGCAGGGCTCAACTCTGTATTGCG -3'BHQ1) (2). Cycle threshold (Ct) values were used to calculate *Fn* copies/total ng fecal DNA using logarithmic regression analysis of a standard curve. The standard was generated using genomic DNA extracted from strain Fn146CP, serially diluted to produce a standard *Fn* copies/ul based on a genome size of 2174500bp. Colonization data is presented as *Fn* copies per ng fecal DNA. A detection limit was set at 100 copies *Fn*/ng DNA because this was the standard concentration at which a Ct value <40 was consistently observed across all assays.

Tissue Harvesting

Mice were euthanized and colons harvested at indicated timepoints. Whole colons were flushed with PBS, measured, and then the final 3 cm (distal colon) was either snap-frozen and stored at -80°C (for gene expression assays) or fixed in 10% formalin. For fluorescent *in situ* hybridization analyses of the mucus layer and luminal microbiome, unflushed colons were fixed in modified Carnoy’s solution (60% methanol, 30% acetic acid, and 10% chloroform) (3). For tumorigenic assessment, whole colons were flushed, cut longitudinally, pinned flat, and fixed in 10% buffered formalin. After formalin fixation, colons were stained with methylene blue for quantification of macroadenomas before being rolled and processed for histopathology assessment (4).

Gene Expression

Snap-frozen distal colons were homogenized in Trizol, and RNA isolated with chloroform phase separation and precipitation with isopropyl alcohol. Isolated RNA was treated with DNAse I (Sigma Aldrich) and then reverse-transcribed to generate cDNA (High Capacity RNA-to-cDNA kit, Applied Biosystems). For 2-week experiments, relative quantification of gene expression was performed with qRT-PCR using Taqman Gene Expression Assays for each target gene, normalized to murine GAPDH (Thermo Fisher Scientific) per sample. After data for all samples were collected, replicate Ct values per mouse were averaged and represented as the relative expression (2^-ddCt^) normalized to control mice. For 11-week experiments, RNA was pre-amplified using a custom pool and then relative quantification of gene expression was performed with qRT-PCR using custom 48-target Taqman Array Plates (Thermo Fisher Scientific), with each gene normalized to murine GAPDH and GUSB. After data for all samples were collected, replicate Ct values per mouse were averaged and represented as the relative expression (2^-ddCt^) normalized to control mice. Gene names, taqman assay IDs, 2^-ddCt^ values, and p-values are displayed in full in **Table S2**.

Histology

Distal colons were fixed in 10% formalin, processed, paraffin-embedded, sectioned, and stained by hematoxylin and eosin (H&E) by the Oncology Tissue Services Core at Johns Hopkins Medical Institutions. Histological assessments of inflammation and tumorigenesis were performed by a blinded pathologist.

Fluorescence In-situ Hybridization (FISH)

Mouse distal colons were fixed in modified Carnoy’s solution with an intraluminal fecal pellet intact when possible prior to embedding in paraffin. 5 mm tissue sections were assessed by FISH using the universal all-bacterial probe (EUB338; 5’- GCTGCCTCCCGTAGGAGT-3’) as well as a *Fusobacterium* specific probe (Fus714; 5’- GGCTTCCCCATCGGCATT-3’), and DAPI counterstain to assess colonization and biofilm presence as previously described (3). Confocal images of FISH-stained mouse colons were taken on a Zeiss LSM 780 META laser scanning microscope at 40X with LSM Zen imaging software in the Johns Hopkins University School of Medicine Microscope Core Facility, then merged in ImageJ.

Whole Genome Sequencing

Whole genome sequencing was done at the PennCHOP Microbiome Center (University of Pennsylvania) after extraction of DNA directly from microbial cultures. Microbial culture samples were shaken at 20000 xg for 2 minutes, then 20 ul of supernatant was taken from each sample tube and added to its corresponding well containing 30 ul of nuclease-free water in a 96 well PCR plate. Extraction cleanup was completed using SPRI/AmpureXP beads in 50% PEG 8000 solution. DNA was then quantified using the Quant-iT^TM^ PicoGreen^TM^ dsDNA assay kit (Thermo Fisher Scientific) before library generation. Shotgun libraries were generated from 0.2ng/ul DNA using the Illumina DNA Prep kit (formerly NexteraFLEX Library Prep kit) and IDT for Illumina unique dual indexes at 1:4 scale reaction volume. Library success was assessed by Quant-iT^TM^PicoGreen^TM^ dsDNA assay after Ampure Cleanup of FLEX libraries using SPRI/AmpureXP beads in 50% PEG 8000 solution. Samples with library yields < 1 ng/ul were re-prepped as needed. Library quality control was assessed through Fragment Analysis using High Sensitivity NGS Fragment Kit. All library samples were then pooled at an equal volume. The library pool was QCed on the Agilent BioAnalyzer to check the size distribution and absence of additional adaptor fragments. This QC pool was then sequenced using a 300 cycle Nano kit on the Illumina MiSeq. Libraries were then repooled based on the demultiplexing statistics of the MiSeq Nano run. Final repooled libraries went through cBot clustering using HiSeq PE Cluster Kit v4 Box 1 of 2 prior to high throughput sequencing using 250 cycle HiSeq SBS Kit v4 Boxes 1 of 2 and 2 of 2 as well as HiSeq PE Cluster Kit v4 – cBot Box 2 of 2 on the Illumina HiSeq. Extraction blanks and nucleic acid-free water were processed along with experimental samples to empirically assess environmental and reagent contamination.  A laboratory-generated mock community consisting of DNA from Vibrio campbellii and Lambda phage were included as a positive sequencing control.

**Supplemental References**

1. Boutaga K, van Winkelhoff AJ, Vandenbroucke-Grauls CM, Savelkoul PH. Periodontal pathogens: a quantitative comparison of anaerobic culture and real-time PCR. FEMS Immunol Med Microbiol. 2005 Aug;45(2):191-9.

2. Drewes JL, White JR, Dejea CM, Fathi P, Iyadorai T, Vadivelu J, et al. High-resolution bacterial 16S rRNA gene profile meta-analysis and biofilm status reveal common colorectal cancer consortia. NPJ Biofilms Microbiomes. 2017;3:34.

3. Dejea CM, Wick EC, Hechenbleikner EM, White JR, Mark Welch JL, Rossetti BJ, et al. Microbiota organization is a distinct feature of proximal colorectal cancers. Proc Natl Acad Sci U S A. 2014 Dec;111(51):18321-6.

4. Wu S, Rhee KJ, Albesiano E, Rabizadeh S, Wu X, Yen HR, et al. A human colonic commensal promotes colon tumorigenesis via activation of T helper type 17 T cell responses. Nat Med. 2009 Sep;15(9):1016-22.
